# Genomic Prediction Within and Across Biparental Families: Means and Variances of Prediction Accuracy and Usefulness of Deterministic Equations

**DOI:** 10.1534/g3.117.300076

**Published:** 2017-09-15

**Authors:** Pascal Schopp, Dominik Müller, Yvonne C. J. Wientjes, Albrecht E. Melchinger

**Affiliations:** * Institute of Plant Breeding, Seed Science and Population Genetics, University of Hohenheim, 70599 Stuttgart, Germany; † Animal Breeding and Genomics, Wageningen University and Research, 6700 AH, The Netherlands

**Keywords:** genomic prediction, biparental families, plant breeding, GBLUP, deterministic accuracy, linkage disequilibrium, GenPred, Shared Data Resources, Genomic Selection

## Abstract

A major application of genomic prediction (GP) in plant breeding is the identification of superior inbred lines within families derived from biparental crosses. When models for various traits were trained within related or unrelated biparental families (BPFs), experimental studies found substantial variation in prediction accuracy (PA), but little is known about the underlying factors. We used SNP marker genotypes of inbred lines from either elite germplasm or landraces of maize (*Zea* *mays* L.) as parents to generate *in silico* 300 BPFs of doubled-haploid lines. We analyzed PA within each BPF for 50 simulated polygenic traits, using genomic best linear unbiased prediction (GBLUP) models trained with individuals from either full-sib (FSF), half-sib (HSF), or unrelated families (URF) for various sizes ($N_{train}$) of the training set and different heritabilities ($h^{2}).$ In addition, we modified two deterministic equations for forecasting PA to account for inbreeding and genetic variance unexplained by the training set. Averaged across traits, PA was high within FSF (0.41–0.97) with large variation only for $N_{train}<50$ and $h^{2}$ <0.6. For HSF and URF, PA was on average ∼40–60% lower and varied substantially among different combinations of BPFs used for model training and prediction as well as different traits. As exemplified by HSF results, PA of across-family GP can be very low if causal variants not segregating in the training set account for a sizeable proportion of the genetic variance among predicted individuals. Deterministic equations accurately forecast the PA expected over many traits, yet cannot capture trait-specific deviations. We conclude that model training within BPFs generally yields stable PA, whereas a high level of uncertainty is encountered in across-family GP. Our study shows the extent of variation in PA that must be at least reckoned with in practice and offers a starting point for the design of training sets composed of multiple BPFs.

With the advent of low-cost genome-wide SNP markers, genomic prediction (GP, see Supplemental Material, Table S1 in File S1 for full list of abbreviations) proposed by Meuwissen *et al.* (2001) has become a powerful tool in animal and plant breeding. The basic idea of GP is to combine the phenotypic and genotypic data of training individuals in a model for predicting the genetic merit of selection candidates that have only been genotyped. Complementing, or even replacing phenotyping can result in considerable cost savings and shortening of breeding cycles, thereby giving GP a big advantage over traditional selection methods (Bernardo and Yu 2007; Goddard and Hayes 2007; Lin *et al.* 2014). Particular challenges of GP in plant breeding arise from (i) the specific population structures mostly characterized by multiple related or unrelated segregating biparental families (BPFs) derived from crosses between inbred parents, and (ii) small samples sizes available for model training (Jannink *et al.* 2010).

In commercial breeding of line and hybrid cultivars, up to several hundred BPFs are newly generated every year. Depending on the species and size of the breeding program, each family can comprise a variable number (usually <250) of lines, developed either by recurrent selfing or the doubled-haploid (DH) technology (Albrecht *et al.* 2011). Since expected differences among BPFs can be reliably predicted based on the mean performance of their parents (Melchinger 1987), GP applied to populations comprising multiple BPFs aims primarily at the identification of superior lines within these families (Riedelsheimer *et al.* 2013). Prediction models such as genomic best linear unbiased prediction (GBLUP) allow capturing Mendelian sampling—responsible for variation in the breeding values of siblings within BPFs—through cosegregation of SNP markers with quantitative trait loci (QTL) (Habier *et al.* 2013). While several studies have investigated the accuracy of GP within and across BPFs, more attention is needed to assess the mean and variation of PA for training sets taken from full-sib (FSF), half-sib (HSF) or unrelated families (URF). Experimental results available so far are confined by the number and size of BPFs (Riedelsheimer *et al.* 2013; Lehermeier *et al.* 2014) and low marker density (Jacobson *et al.* 2014; Lian *et al.* 2014).

Model training with individual BPFs has been studied intensively, and PA has been generally more promising for “within-family GP” than “across-family GP” (Riedelsheimer *et al.* 2013). Various authors argued that for a given size of the training set, within-family GP would provide the highest possible PA owing to strong linkage disequilibrium (LD) between SNPs and QTL due to cosegregation and the same set of loci being polymorphic in the prediction and training set (Crossa *et al.* 2014; Lehermeier *et al.* 2014). Nevertheless, Lian *et al.* (2014) reported for within-family GP substantial variation in PA among 969 BPFs and various traits, in line with the results of other studies on BPFs (Riedelsheimer *et al.* 2013; Jacobson *et al.* 2014; Lehermeier *et al.* 2014). However, a systematic investigation on the extent and factors determining the mean and variation in PA among BPFs and traits is, to the best of our knowledge, not available to date.

Since PA increases with closer pedigree relationships between training and predicted individuals (Habier *et al.* 2010; Clark *et al.* 2012), one obvious strategy is to use HSFs with one common parent between the training family (BPF_train_) and the predicted family (BPF_pred_) in across-family GP. Compared to within-family GP, PA for this strategy was generally much lower with the same sample size, but can reach similar levels if the sample size is strongly extended (Lehermeier *et al.* 2014). By comparison, model training with only unrelated BPFs produced from the same ancestral population yields often poor or even negative PA (Riedelsheimer *et al.* 2013; Jacobson *et al.* 2014; Schopp *et al.* 2017). Optimizing training set designs in GP with BPFs therefore requires better insights into how the pedigree relationship between BPFs, the sample size, and the heritability affect the mean and the variation in PA. Herein, we address these factors for the simple case of GP across individual pairs of BPFs, thereby providing a starting point for further investigations on the design of multi-family training sets in plant breeding.

Forecasting PA based on existing molecular and phenotypic data could assist breeders in (i) choosing the most suitable BPFs for model training for prediction of existing or planned BPFs, and (ii) allocating resources to the training and prediction sets. Daetwyler *et al.* (2008, 2010) derived a deterministic equation for forecasting PA, which requires only population parameters (sample size $N_{train},$ heritability $h^{2},$ and the effective number of chromosome segments $M_{e}).$ When averaged over several traits, empirical and deterministic accuracy agreed well within BPFs (Lorenz 2013; Riedelsheimer *et al.* 2013; Lian *et al.* 2014). There is little consensus, however, regarding the calculation of $M_{e}\;$in general (Goddard 2009; Meuwissen and Goddard 2010; Goddard *et al.* 2011; Wientjes *et al.* 2013), and, specifically, for BPFs (Lorenz 2013; Riedelsheimer and Melchinger 2013; Lian *et al.* 2014). Recently, Daetwyler’s equation was applied to both GP within and across cattle breeds (Wientjes *et al.* 2013, 2015). The authors extended Goddard *et al.*’s (2011) approach for calculating $M_{e}$ from the variance of genomic relationship coefficients to multiple populations. Overestimation of PA was attributed to a violation of Daetwyler’s assumption that the genetic variance in the prediction set is fully explained by marker effects estimated in the training set. An aggravation of this problem is expected for across-family GP with BPFs due to a high fraction of QTL and markers that are not consistently polymorphic across BPFs. Herein, we propose to extend Daetwyler’s equation to cope with this problem and make the equation applicable to across-family GP in plant breeding.

Alternatively, PA can be forecasted based on the estimated reliability of genomic-estimated breeding values (GEBVs) derived from selection index theory (VanRaden 2008). However, this approach has rarely been applied in plant breeding (Akdemir *et al.* 2015; He *et al.* 2016), and, to the best of our knowledge, not to GP of individual BPFs, despite promising results for GP within and across breeds of cattle (Hayes *et al.* 2009; Wientjes *et al.* 2013, 2015). One problem is that the approach was developed for outbred populations, and needs modifications when applied to inbred genotypes. Moreover, several strict assumptions regarding the properties of the genomic relationship matrix must be satisfied to obtain meaningful results, which will be elaborated in this paper for the case of BPFs in plant breeding.

The objectives of our study were to (i) investigate the mean and variation of empirical PA within and across BPFs of inbred lines, (ii) examine how the variation in PA is affected by differences in polymorphism at causal loci of polygenic traits between the training and prediction set, as well as by other factors (*e.g.*, level of ancestral LD, pedigree relationship between BPFs, sample size, heritability), and (iii) adapt equations for deterministic forecasting of PA in BPFs of inbred genotypes and demonstrate their usefulness in simulated data sets. To simulate realistic scenarios, we used SNP data of inbred lines taken either from a public maize breeding program or a DH library of a European maize landrace and generated *in silico* numerous BPFs of DH lines. Besides flexibility in the choice of sample sizes, and exclusion of nuisance factors uncontrollable in experimental studies, this allowed us to simulate traits with known genetic architecture for a profound analysis of the causal factors affecting PA of GP within and across BPFs.

## Materials and Methods

### Ancestral populations

We considered two ancestral populations as source germplasm of parental genotypes for generating BPFs. Ancestral population *Elite* consisted of 72 elite inbred lines with medium long-range LD (Figure S1A in File S1) representative for the Flint heterotic group of the maize breeding program of the University of Hohenheim. Ancestral population *Landrace* consisted of 40 DH lines derived without any intentional selection from the German maize landrace “Gelber Badischer” with a rapid decay of LD to a low level (Melchinger *et al.* 2017). All lines were genotyped with the Illumina chip MaizeSNP50, containing 57,841 SNPs, and were expected to be fully homozygous. Markers monomorphic in the ancestral population or heterozygous in at least one individual were removed for further analysis. Physical map positions were converted into genetic map positions required for simulating meioses as described by Schopp *et al.* (2017). In total, we retained 19,204 and 16,171 SNPs for *Elite* and *Landrace*, respectively, distributed over the 10 maize chromosomes ranging in length from 137 to 276 cM (1913 cM in total). Individuals in the ancestral population were regarded as unrelated for defining pedigree relationships between subsequently generated BPFs.

### Simulation of BFPs

For generating BPFs, we first sampled at random $N_{P}$ = 25 parent lines from each ancestral population, and intermated them according to a half-diallel design to generate all $\left(\begin{array}{c} N_{P}\\ 2 \end{array}\right)=300$ possible crosses. Subsequently, 1500 DH lines were derived from each F_1_ cross to obtain the BPFs used for further analyses. According to the half-diallel, each predicted family (BPF_pred_ =A) was associated with several possible training families (BPF_train_ =B) with different pedigree relationships to A. These were: one FSF, corresponding to A=B; $2(N_{P}-2)=46$ HSF B, sharing one common parent with A; and (iii) $\left(\begin{array}{c} N_{P}\\ 2 \end{array}\right)-2\left(N_{P}-2\right)-1=253$ URF B, sharing no common parent with A. Meioses for *in silico* production of DH lines were simulated with the *R* package *Meiosis* (Müller and Broman 2017).

### Description of factors analyzed

For systematic assessment of the factors influencing the distribution of the empirical PA, we defined various fixed and random factors (Table 1). As fixed factors, we considered (i) the ancestral population (*Elite* or *Landrace*), (ii) the pedigree relationship (FSF, HSF, or URF) between individuals in BPF_pred_ and BPF_train_, (iii) the type of data (SNP marker genotypes or QTL genotypes) used to calculate the genomic relationship matrix G for GBLUP, (iv) the sample size $N_{train}=25,100,250$, and (v) the heritability of the trait $h^{2}=0.3,0.6,1.0.$ The idealistic scenario $h^{2}=1$ was included to demonstrate how the variation in PA behaves when phenotypic accuracy is not a limiting factor. Random factors were the trait T, the BPF_pred_ A, the BPF_train_ B, as well as the actual sample of training individuals R taken from B.

**Table 1:** Overview of factors with their corresponding levels analyzed in this study

Type	Factor	Model Parameter	Number of Factor Levels	Factor Levels
Fixed factors	Ancestral population	—	2	**Elite**, Landrace
	Pedigree relationship between training and predicted family	—	3	**FSF**, **HSF**, **URF**
	Data used to calculate the relationship matrix	—	2	QTL, **SNPs**
	Sample size ($N_{train}$)	—	3	25, **100**, 250
	Heritability ($h^{2}$)	—	3	0.3, **0.6**, 1
Random factors	Trait	T	50	—
	Predicted family (BPF_pred_)	A	50	—
	Training family (BPF_train_)	B	1 (FSF), 25 (HSF/URF)	—
	Training set sample	R	3	—

Default values for the standard scenario are indicated in boldface.

We simulated 50 truly polygenic traits T = 1,…,50, each governed by 1000 QTL. First, we sampled at random a subset of 5000 SNP markers from all SNPs available in the ancestral population, corresponding to a marker density of 2.61 SNPs cM^−1^. This fixed set of marker was used for GP of all traits, because resampling of SNP marker positions had a negligible influence on the results. Second, for each of the 50 traits we sampled at random the map positions of 1000 QTL from the remaining 14,204 and 12,171 SNPs in *Elite* and *Landrace*, respectively. Following Meuwissen *et al.* (2001), effects of each QTL were drawn from a Gamma distribution Γ(0.4,1.66) with equal probability of effect signs. Importantly, all traits were affected by the same number of loci, but differed in the position and effects of QTL. Thus, the realized number of polymorphic QTL loci could vary depending on the trait and the BPF_pred_ and BPF_train._

Phenotypes y of training individuals were simulated according to the model y=g+e (*cf*. Goddard *et al.* 2011), where g is the vector of true breeding values (TBVs) calculated as g=Wa, W is the matrix of genotypic scores at QTL coded as 2 or 0, depending on whether a DH line was homozygous for the 1 or 0 allele, respectively, and a is the vector of QTL effects. Vector e contains independent normally distributed environmental noise variables, where variance $\sigma_{e}^{2}$ was assumed to be constant across BPFs derived from one ancestral population, implying independent environmental influence on the phenotypes. We calculated $\sigma_{e}^{2}=\left(h^{2}\bar{\sigma_{g}^{2}}-\bar{\sigma_{g}^{2}}/h^{2}\right),$ where $h^{2}$ is the *a priori* specified heritability (*cf*. Table 1) and $\bar{\sigma_{g}^{2}}$ is the genetic variance within a BPF, averaged across all 300 BPFs and 50 traits simulated.

Finally, we sampled at random 50 out of the 300 BPFs, and considered them individually as the predicted family BPF_pred_ A. From the 1500 DH lines in each BPF_pred_, we estimated GEBVs for the first 500 lines. For within-family GP, training individuals were sampled from the remaining 1000 lines to predict individuals within the same family (A=B, FSF). For across-family GP (A≠B, HSF or URF), 25 BPF_train_ serving individually for model training were sampled from the 46 available HSFs and the 253 available URFs, respectively. For given BPF_pred_ and BPF_train_, we sampled from BPF_train_ three disjunct samples R of individuals of size $N_{train}$ (according to the fixed factor “sample size,” Table 1) with which the prediction model was trained. To minimize variation in PA attributable to sampling individuals from the BPF_pred_, we chose $N_{pred}=500.$ By contrast, the numbers $N_{train}$ were of realistic magnitude, and analyzing repeated samples allowed us to quantify the variation in PA due to finite sampling in BPF_train_.

### Genomic prediction model

The GBLUP model can be written as y=1μ+Zu+ε, where μ is the general mean, Z is an incidence matrix linking phenotypes with breeding values, u is the vector of random breeding values with mean zero and variance-covariance matrix var(u)=G∘Σ, where G is the genomic relationship matrix and $\Sigma =\left[\begin{array}{cc} J_{\mathrm{AA}}\sigma_{u,A}^{2} & J_{\mathrm{BA}}\sigma_{u,A}\sigma_{u,B}r_{AB}\\ J_{\mathrm{BA}}\sigma_{u,A}\sigma_{u,B}r_{AB} & J_{\mathrm{BB}}\sigma_{u,B}^{2} \end{array}\right],$ $\sigma_{u,A}^{2}\;$ and $\sigma_{u,B}^{2}$ are the additive variances in the noninbred reference population of BPF_pred_ and BPF_train_, respectively, which correspond to their (outbred) F_2_ generation. $J_{AA},$ $J_{BB}$ and $J_{AB}=J_{BA}$ are matrices of 1’s,$\;r_{AB}$ is the genetic correlation between populations A and B, which was assumed to be equal to 1 for reasons detailed in the discussion, and ∘ symbolizes the Hadamard product. Vector ε contains random residuals with mean zero and $\mathrm{var}\left(\varepsilon \right)=I\sigma_{\varepsilon }^{2},$ where I is an identity matrix and $\sigma_{\varepsilon ,B}^{2}$ is the residual error variance. We used $G=\left[\begin{array}{cc} G_{AA} & G_{AB}\\ G_{BA} & G_{BB} \end{array}\right],$ representing a modified version of the block-structured genomic relationship matrix devised by Chen *et al.* (2013), where the across-population blocks $G_{AB}=G_{BA}^{T}$ had elements

$$G_{A_{i},B_{j}}=\frac{\sum_{k}\left(x_{A_{i},k}-2p_{A,k}\right)\left(x_{B_{j},k}-2p_{B,k}\right)}{\sqrt{2\sum_{k}p_{A,k}\left(1-p_{A,k}\right)}\sqrt{2\sum_{k}p_{B,k}\left(1-p_{B,k}\right)}},$$ (1)

and $x_{A_{i},k}$ and $x_{B_{j},k}$ are the genotypic scores of DH lines i and j in population A and B at locus k, respectively, coded as 2 and 0, and $p_{A,k}$ and $p_{B,k}\;$are the allele frequencies at locus k in A and B, respectively, where k=1,…,1,000 or k=1,…,5,000 depending on whether QTL or SNPs were used to calculate G (according to the fixed factor “data,” Table 1). Submatrices $G_{AA}$ and $G_{BB}$ are calculated accordingly, but here the denominator simplifies to $2\sum_{k}p_{A,k}\left(1-p_{A,k}\right)$ and $2\sum_{k}p_{B,k}\left(1-p_{B,k}\right),$ respectively, corresponding to the standard G matrix without subpopulation structure (Habier *et al.* 2007; VanRaden 2008). Importantly, the denominator for matrix $G_{AB}$ in Equation 1 is different from that in Chen *et al.* (2013), who used $2\sum_{k}\sqrt{p_{A,k}\left(1-p_{A,k}\right)p_{B,k}\left(1-p_{B,k}\right)}.$ Their approach effectively removes all loci that are monomorphic in A and/or B, whereas our denominator retains these loci in the scaling of G, yielding a better approximation of the true relationship matrix, as discussed below.

In any BPF derived from fully homozygous parents, the expected allele frequency of a locus is known to be either 0, 0.5, or 1, depending on the genotypes of the parents. These expected frequencies were used in the computation of genomic relationships. Since, in our study, only population B had phenotypes, we used a single-group GBLUP model. Although we allowed for heterogeneous genetic variances among BPFs in the general model (Equation 1) and the derivation of reliability described below (see Appendix B), $\sigma_{u,A}^{2}$ enters the computation of GEBVs in A as a constant factor (see Equation B4) and, hence, does not affect the empirical PA. Estimates $\hat{\sigma_{u,B}^{2}}$ and $\hat{\sigma_{\varepsilon ,B}^{2}}$ for BPF_train_ were obtained by restricted maximum likelihood from the $N_{train}$ individuals in the training set using the *mixed*.*solve* function from R-package *rrBLUP* (Endelman 2011). The empirical PA was calculated as the correlation between GEBVs $\hat{u}$ and the TBVs u for the 500 predicted individuals in BPF_pred_.

### Analysis of variance of empirical prediction accuracies

For each possible combination of fixed factors (*cf*. Table 1), we partitioned $\sigma_{total}^{2},$ the total variance of the empirically observed PA $\rho_{ABRT},$ into variance components caused by each random factor, where we assumed a hierarchical structure for BPF_pred_ A, BPF_train_ B and the training set sample R, as well as cross-classification with factor trait T. Estimates of the variance components were obtained from the following random-effects model using function *lmer* of *R* package *lme4* (Bates *et al.* 2015):

$$\rho_{ABRT}=\mu +A+A:B+A:B:R+T+A\times T+\left(A:B\right)\times T+\left(A:B:R\right)\times T,$$ (2)

where μ is the overall mean of PA for each of the three pedigree relationships (FSF, HSF, and URF) between individuals in A and B analyzed; A is the effect of the BPF_pred_; A:B is the effect of the BPF_train_ B nested within A; A:B:R is the effect of the rth sample of training individuals from B nested within A; *T* is the effect of the trait, A×T is the interaction effect of BPF_pred_ A with trait T; (A:B)×T is the interaction effect of BPF_train_ B nested within A with trait T; and (A:B:R)×T is the interaction effect of the training set sample nested within B:A with trait T, which corresponds to the residual error of the model. In the case of FSF (A=B), all random factors involving B were dropped. The degrees of freedom for each factor are shown in Table S2 in File S1.

### Deterministic equations for forecasting prediction accuracy (PA)

We followed the theoretical framework of Wientjes *et al.* (2015) for forecasting PA within and across populations using two deterministic equations. Both equations assume that actual relationships regarding QTL are known, and were originally developed for outbred individuals. Hence, modifications are required to apply the equations to inbred individuals. As mentioned above, the outbred reference population corresponding to a BPF of fully inbred (DH) lines with an inbreeding coefficient of F=1 is the F_2_ generation. The level of inbreeding in BPFs of DH lines is reflected in the diagonal elements of G calculated according to Equation 1, yielding $G_{ii}=1+F_{i}=2$ in the special case of BPFs derived from homozygous parents.

The first approach is based on the reliability of GEBVs of each individual in A (VanRaden 2008; Wientjes *et al.* 2013, 2015). Using the formula for the reliability of a selection index given by Mrode (2005, p. 15) and replacing the genetic covariance matrices by the genomic relationship matrices [multiplied by the corresponding genetic (co)variance components] yields the following formula that accounts for inbreeding in the predicted individual (see Appendix B):

$$r_{A_{i}}^{2}=r_{AB}^{2}\frac{G_{A_{i},B}{}^{T}{\left[G_{BB}+R\frac{\hat{\sigma_{\varepsilon ,B}^{2}}}{\hat{\sigma_{u,B}^{2}}}\right]}^{-1}G_{A_{i},B}}{G_{A_{i},A_{i}}},$$ (3)

where $r_{AB}^{2}$ is the squared genetic correlation between A and B (here $r_{AB}^{2}=1$), $G_{A_{i},B}$ is the vector of genomic relationships of individual i in A with all training individuals of B, R=I is an identity matrix when assuming independent residual error variances, and $G_{A_{i},A_{i}}$ is the relationship of individual i∈A with itself, providing an estimate of $1+F_{i}.$ Dividing by $G_{A_{i},A_{i}}$ assures that reliabilities are correctly scaled, given that variance components and inbreeding refer to an outbred reference population, as is the case when calculating G according to Equation 1 (see Appendix B). The deterministic PA in population A was subsequently obtained by averaging over all individuals in A as $\rho^{W}=\sqrt{1/N_{A}\sum_{i=1}^{N_{A}}r_{A_{i}}^{2}},$ where in our case $N_{A}=500.$

The second equation was proposed by Daetwyler *et al.* (2008, 2010) and is based solely on population parameters, which was modified to account for unexplained variance in A by accounting for different markers segregating in A and B (in cases where A≠B):

$$\rho^{D}=\sqrt{\theta_{AB}\frac{N_{train}h^{2}}{N_{train}h^{2}+M_{e}},}$$ (4)

with $\theta_{AB}=\left|L_{A\cap B}\right|/\left|L_{A}\right|,$ where $\left|L_{A\cap B}\right|$ is the number of markers that segregate in both A and in B and $\left|L_{A}\right|$ is the number of markers that segregate in A, $N_{train}$ is the sample size, $h^{2}=\left(1+F_{B}\right)\hat{\sigma_{u,B}^{2}}/\left[\left(1+F_{B}\right)\hat{\sigma_{u,B}^{2}}+\hat{\sigma_{\varepsilon ,B}^{2}}\right],$ where $F_{B}=\bar{diag\left(G_{BB}\right)}-1$ is the average inbreeding coefficient of the individuals in B, $\hat{\sigma_{u,B}^{2}}$ refers to the estimated additive variance in the (outbred) F_2_ generation of B, and $M_{e}$ is the effective number of chromosome segments. Wientjes *et al.* (2015) proposed an estimator for $M_{e}$ across outbred populations, which is calculated as

$$M_{e}=\frac{1}{\mathrm{var}\left[G_{A_{i},B_{j}}-E\left(G_{A_{i},B_{j}}\right)\right]},$$ (5)

where $G_{A_{i},B_{j}}$ contains all genomic relationships between individuals i from A and training individuals j from B. Given a uniform pedigree relationship between individuals in A and B (*e.g.*, FSF, HSF, and URF), the denominator simplifies to $\mathrm{var}\left(G_{A_{i},B_{j}}\right),$ because $\text{E}\left(G_{A_{i},B_{j}}\right)=\text{constant}.$ If the individuals i from A and j from B have inbreeding coefficients $F_{A}$ and $F_{B},$ respectively, we propose to use (*see* Appendix C):

$$M_{e}=\frac{\left(1+F_{A}\right)\left(1+F_{B}\right)}{\mathrm{var}\left(G_{A_{i},B_{j}}\right)}.$$ (6)

For DH lines from BPFs, $1+F_{A}=\bar{diag\left(G_{AA}\right)}=2$ and $1+F_{B}=\bar{diag\left(G_{BB}\right)}=2,$ so that $M_{e}=4/\mathrm{var}\left(G_{A_{i},B_{j}}\right),$ which was herein used as estimator for $M_{e}.$

### Comparison of empirical and deterministic prediction accuracies

For all analyses except the ANOVA of $\rho_{ABRT},$ we considered only one sample R of training individuals and dropped index R altogether. This simplifies the presentation of our results and corresponds to the realistic case of having only one specific sample of training individuals available. For comparison of PA between fixed factors (*e.g.*, between samples sizes, heritabilities or ancestral populations), as well as for evaluating the overall agreement of empirical and deterministic PAs, we calculated the general mean of PA across all random factors A, B, and T, subsequently denoted as $\bar{\rho },$ $\bar{\rho^{W}},$ and $\bar{\rho^{D}}$ for the empirical PA and the two deterministic PAs, respectively.

### Causal analysis of the variation in PA among traits in GP across BPFs

Preliminary analyses showed that PA varied substantially among traits in across-family GP for HSFs and URFs, although we assumed the same polygenic architecture for all 50 simulated traits. Therefore, we devised additional simulations to investigate the underlying cause(s), using assumptions warranting almost ideal conditions for GP to largely eliminate the influence of nuisance factors on PA. We restricted these simulations to HSFs to demonstrate the key points in a simple fashion. First, we chose at random (i) a pair of HSFs BPF_pred_ A and BPF_train_ B produced from ancestral population *Elite*, and (ii) repeatedly sampled 1000 QTL positions from the entire set of 19,204 SNPs until we found a sample with $\theta_{AB}\approx 0.4,$ corresponding to the average value of $\theta_{AB}$ for HSF in our study (Table 2). Second, given A and B and the 1000 QTL positions, we sampled 1000 sets of different QTL effects $a_{k}$ as described above. This resulted in 1000 traits with $\theta_{AB}\approx 0.4$ and identical QTL positions, but different QTL effects. Finally, assuming $N_{train}=250,h^{2}=1$ and known QTL genotypes, we used RR-BLUP—yielding equivalent GEBVs as GBLUP (Habier *et al.* 2007)—to identify among the 1000 traits the two with lowest and highest PA and retrieved the corresponding QTL effect estimates.

**Table 2:** Mean (±SD) of the estimated number of effective chromosome segments ($M_{e}$) and the proportion of polymorphic loci in the predicted family A that also segregate in the training family B ($\theta_{AB}$) with different pedigree relationships (FSF, HSF, and URF) between A and B, derived either from ancestral populations *Elite* or *Landrace*.

Ancestral Population	Pedigree Relationship	$M_{e}$ ± SD	$\theta_{AB}\pm$SD
*Elite*	FSF	21.00 ± 2.27	1.00 ± 0.00
	HSF	66.26 ± 27.03	0.50 ± 0.10
	URF	148.16 ± 77.87	0.40 ± 0.08
*Landrace*	FSF	22.24 ± 2.05	1.00 ± 0.00
	HSF	72.48 ± 24.83	0.50 ± 0.08
	URF	172.33 ± 77.03	0.40 ± 0.06

We surmised that variation in PA among traits arises from structural differences in the large chromosome segments containing cosegregating QTL alleles that DH lines inherit from their respective parents. To investigate this hypothesis, we analyzed the contribution of each chromosome segment along the entire genome to PA. The length of the chromosome segments within A and B was taken as the expected genetic map distance at which the LD between two QTL in BPFs falls below $r^{2}=0.2$ (*cf*. Giraud *et al.* 2014), which amounted to 41 cM (*cf*. File S3 in Schopp *et al.* 2017). Using a sliding window approach, chromosome segments of this length moved in steps of 5 cM along each chromosome separately for each trait. Similar to Kemper *et al.* (2015), we subsequently calculated for each window W the “local” TBV for all DH lines i∈A in the BPF_pred_ as

$$TBV_{i,W}=\sum_{k\epsilon W}x_{i,k}a_{k},$$ (7)

where $x_{i,k}$ is the genotypic score coded (2,0) for DH line i at QTL k∈W, and $a_{k}$ is the corresponding QTL effect. Analogously, we calculated the local GEBV in the BPF_pred_ as

$$GEBV_{i,W}=\sum_{k\epsilon W}x_{i,k}\hat{a_{k}},$$ (8)

where ${\hat{a}}_{k}$ is the estimate of $a_{k}$ obtained from RR-BLUP in BPF_train_ B, provided k segregated in B, and otherwise ${\hat{a}}_{k}=0.$ Subsequently, we calculated for each window W the correlation between local TBVs and local GEBVs among all 500 DH lines in A.

Further, we defined *chromosome segment substitution effects* (CSSE) for the parental chromosome segments of A as the sum of allele substitution effects across all QTL k∈W

$$CSSE_{A,W}=\sum_{k\epsilon W}\delta_{A,k}a_{k}=\frac{1}{2}\left(TBV_{P_{A2},W}-TBV_{P_{A1},W}\right),$$ (9)

where $\delta_{A,k}=\left(x_{i,k,P_{A1}}-x_{i,k,P_{A2}}\right)/2,$ $P_{A1}$ and $P_{A2}$ are the parents of A with$\;P_{A2}$ being the common parent of A and B. Thus, $\delta_{A,k}=\pm 1,$ if $P_{A1}$ and $P_{A2}$ carry different alleles at QTL k, and $\delta_{A,k}=0,$ otherwise. Values $CSSE_{B,W}$ were calculated analogously with respect to parents $P_{B1}$ and $P_{B2}=P_{A2}$ of B. Note that $\delta_{A,k}=\delta_{B,k},$ if QTL k segregates in both A and B, *i.e.*, $P_{A1}$ and $P_{B1}$ carry the same allele that is different from the allele in $\;P_{B2}=P_{A2}.$ In contrast, $\delta_{A,k}\neq \delta_{B,k}$ implies that QTL k segregates in exactly one of the two HSFs A or B. Thus, $CSSE_{A,W}-CSSE_{B,W}\neq 0$ only if $\delta_{A,k}\neq \delta_{B,k}$ at one or more QTL k∈W, and the magnitude of this difference depends on (i) the subset of QTL k∈ W with $\delta_{A,k}\neq \delta_{B,k},$ (ii) the relative size of $a_{k}$ for each QTL in W compared with the effects of other QTL in the genome, and (iii) whether these effects have identical sign or not, which is important, especially for QTL that are closely linked. Altogether, the magnitude of $CSSE_{A,W}$ and its difference to $CSSE_{B,W}$ for each trait along the genome were expected to strongly influence the PA of GEBVs in BPF_pred_ A, estimated on the basis of BPF_train_ B.

All computations were carried out in the *R* statistical environment (R Core Team 2017).

### Data availability

Genotypic data of the ancestral populations is available in File S2. All R packages used for simulating the data are publicly available. All simulation steps and equations are fully described within the manuscript.

## Results

### Means and variation of empirical PA

**Figure 1:**
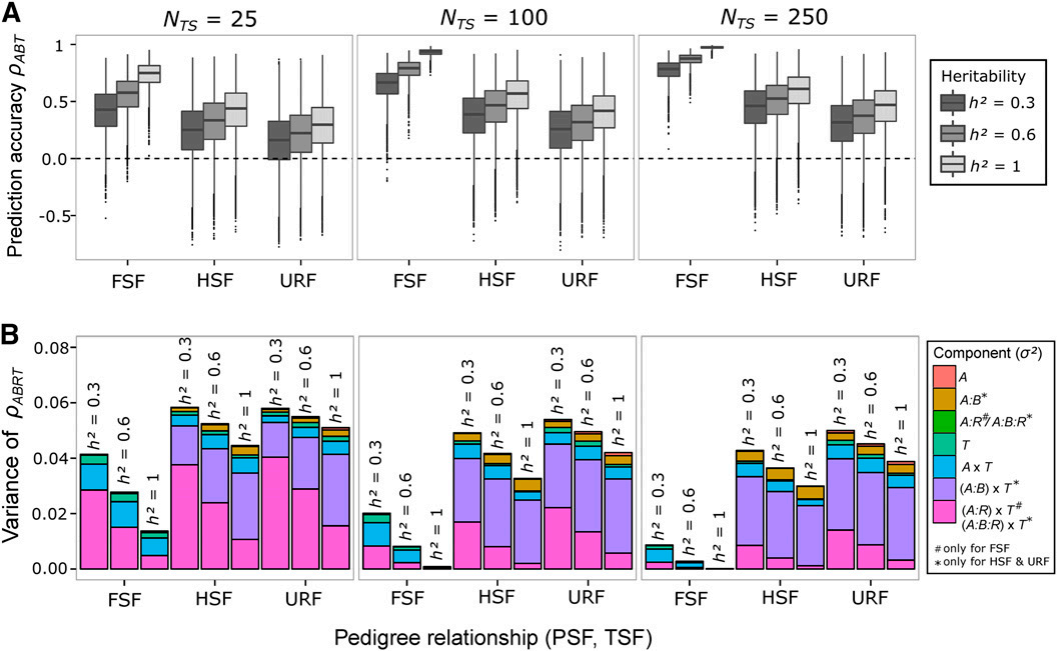
(A) Boxplots of empirical prediction accuracies $\rho_{ABT}$ in BPFs of DH lines, and (B) variance components of different factors influencing the variation of $\rho_{ABRT}.$ Parents of BPFs were sampled from ancestral population *Elite*, and SNP markers were used to calculate the genomic relationship matrix G. Results are shown for different pedigree relationships (FSF, HSF, and URF) between the predicted family (BPF_pred_) A and training family (BPF_train_) B, as well as for different sample sizes $N_{train}\;$and heritabilities $h^{2}.$

Figure 1A shows the distributions of empirical PA $\rho_{ABT}.$ For the standard scenario (ancestral population *Elite*, $N_{train}=100,h^{2}=0.6,$ and G calculated from SNP markers, Table 1), the mean PA ($\bar{\rho }$) across all pairs of BPF_pred_ and BPF_train_ and traits was highest for FSF (0.79, Table S3 in File S1), and decreased by 43% for HSF (0.45) and by 60% for URF (0.32). A reverse trend was observed for the SD of $\rho_{ABT},$ which amounted to 0.09 for FSF and more than doubled for HSF (0.20) and URF (0.22). The 5 and 95% quantiles of $\rho_{ABT}$ ranged from 0.61 to 0.89 for FSF, but from 0.07 to 0.73 for HSF and from −0.09 to 0.64 for URF.

For $h^{2}=0.6,$ reducing $N_{train}$ from 100 to 25 resulted in 28–31% lower $\bar{\rho }$ and increasing $N_{train}$ to 250 resulted in 12–18% higher $\bar{\rho }$ for all pedigree relationships (Figure 1A). The SD increased for $N_{train}=25$ by 84% for FSF, but only by 11 and 4% for HSF and URF, respectively, because it was already large under $N_{train}=100.$ For $N_{TS}=250,$ the SD reduced by 42% for FSF, yet only by 6% for HSF and 4% for URF. Altering $h^{2}$ for $N_{train}=100$ affected the PA similarly as altering $N_{train}$ under fixed $h^{2}.$ In comparison with $h^{2}=0.6,$ $\bar{\rho }$ was reduced by 18–20% for$\;h^{2}=0.3$ and increased by 20–32% for $h^{2}=1.0,$ depending on the pedigree relationship. The corresponding SDs changed considerably for FSF (+57 and −68%), but only marginally for HSF (8 and −11%) and URF (4 and −7%).

**Figure 2:**
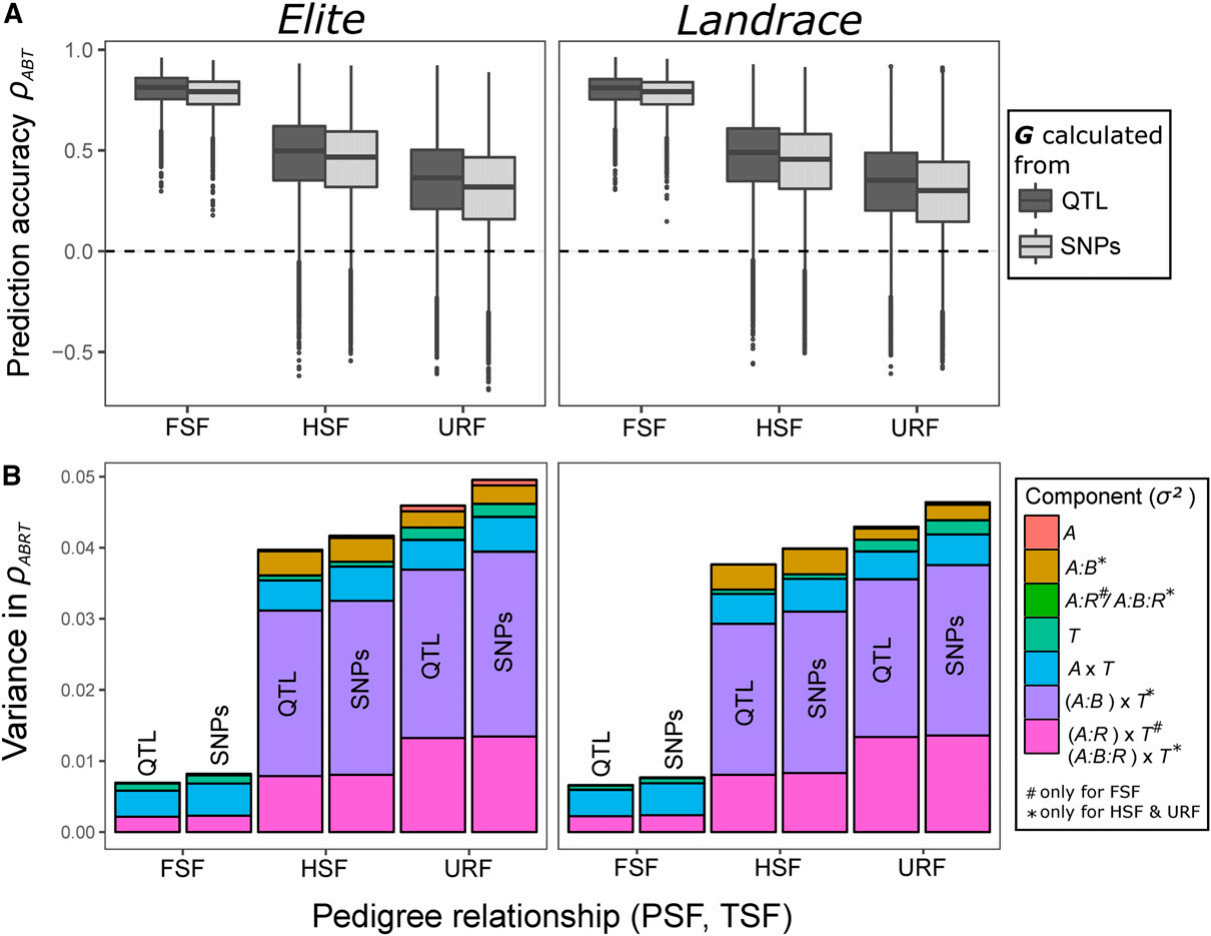
(A) Boxplots of empirical prediction accuracies $\rho_{ABT}$ in BPFs of DH lines and (B) variance components of different factors influencing the variation of $\rho_{ABRT}.$ Parents of BPFs were sampled from ancestral population *Elite* (left) or *Landrace* (right), and either genotypes at SNP markers or at QTL were used to calculate the genomic relationship matrix G. Results are shown for different pedigree relationships (FSF, HSF, and URF) between the predicted family (BPF_pred_) A and training family (BPF_train_) B and refer to $N_{train}=100$ and $h^{2}=0.6.$

Deriving BPFs from ancestral population *Landrace* instead of *Elite* generally reduced $\bar{\rho }$ by <0.05, whereas the SD remained nearly identical (Figure 2A and Table S3 in File S1). By comparison, calculating the G matrix from QTL instead of SNP data increased $\bar{\rho }$ by only 0.02, 0.03, and 0.05 for FSF, HSF, and URF, respectively, but hardly affected the SD, regardless of the pedigree relationship and the ancestral population.

### Analysis of variance of random factors affecting the empirical PA

Estimates of $\sigma_{total}^{2}$ for $\rho_{ABRT}$ were of similar magnitude for HSF and URF, but generally much smaller for FSF (Figure 1B). For the standard scenario, $\sigma_{total}^{2}$ was small for FSF (0.01) and primarily attributable to $\sigma_{A\times T}^{2}.$ By comparison, $\sigma_{total}^{2}$ was 5.3 and 6.6 times larger for HSF and URF, respectively, with >50% contributed by $\sigma_{\left(A:B\right)\times T}^{2},$ followed by the residual variance $\sigma_{\left(A:B:R\right)\times T}^{2}$ (26 and 19%, respectively). All variance components not involving factor T were substantially smaller, with $\sigma_{A:B}^{2}$ contributing most for HSF (9%) and URF (6%).

Decreasing $N_{train}$ to 25 or $h^{2}$ to 0.3 affected the relative importance and overall magnitude of the variance components similarly for the three pedigree relationships (Figure 1B). The residual variances $\sigma_{\left(A:R\right)\times T}^{2}$ (FSF) and $\sigma_{\left(A:B:R\right)\times T}^{2}$ (HSF, URF) increased substantially, accompanied by a moderate increase in $\sigma_{A\times T}^{2}$ for FSF and decrease in $\sigma_{\left(A:B\right)\times T}^{2}\;$for HSF and URF. Conversely, increasing $N_{train}$ to 250 or $h^{2}$ to 1.0 strongly reduced the residual variances and nearly eliminated $\sigma_{total}^{2}$ for FSF, whereas, for HSF and URF, $\sigma_{total}^{2}$ remained large owing to a high $\sigma_{\left(A:B\right)\times T}^{2},$ even under these favorable conditions.

Deriving BPFs from ancestral population *Landrace* instead of *Elite* had almost no effect on $\sigma_{total}^{2}$ and its components (Figure 2B). Calculating the G matrix from QTL instead of from SNP genotypes moderately reduced $\sigma_{total}^{2}$ by 5% for HSF and 10% for URF, mainly due to decreasing $\sigma_{\left(A:B\right)\times T}^{2}.$ In contrast to HSF and URF, $\sigma_{total}^{2}$ for FSF was already minor when using SNP genotypes, leaving less room for improvement when using QTL instead of SNP genotypes than for HSF and URF, which both showed bigger changes in the absolute magnitude of the variance components than FSF.

### Comparison of empirical and deterministic prediction accuracies

**Figure 3:**
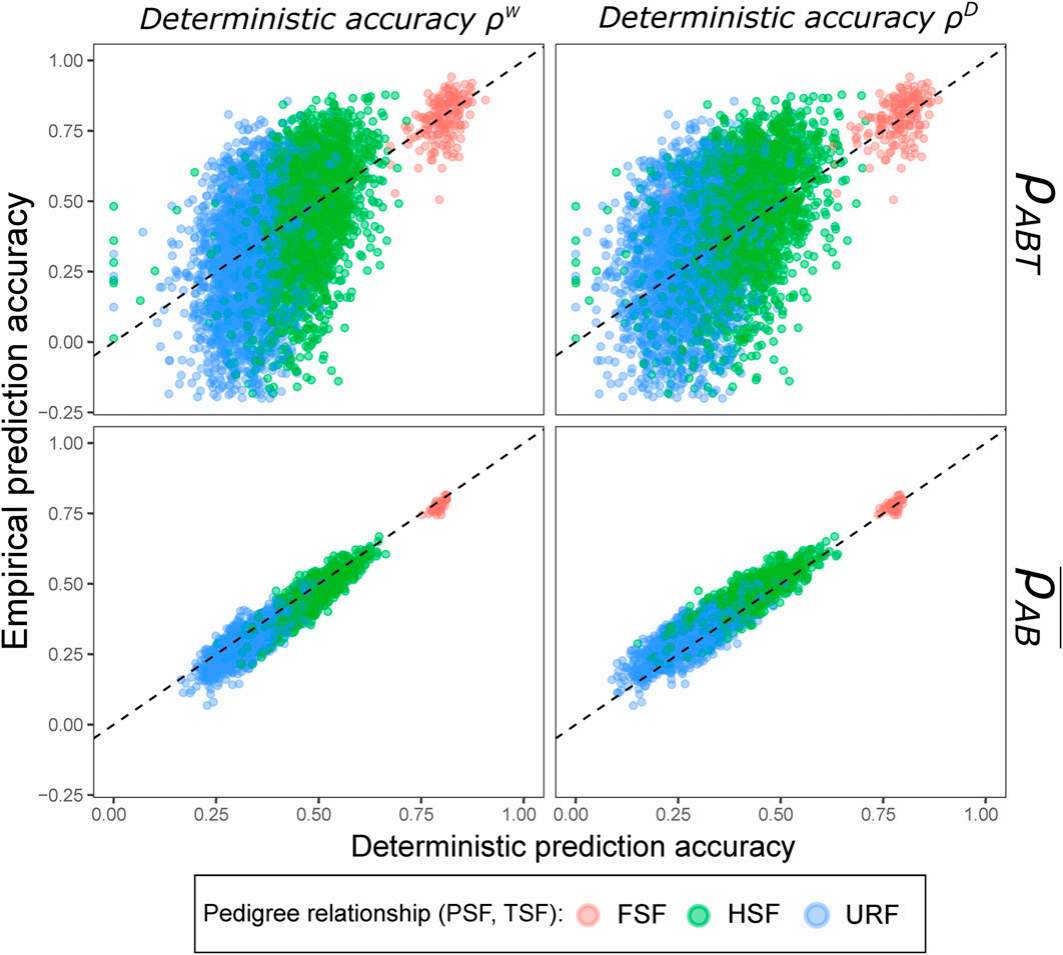
Empirical prediction accuracy ρ in BPFs of DH lines plotted against deterministic prediction accuracies $\rho^{W}$ and $\rho^{D}.$ The top two graphs refer to observations for single traits ($\rho_{AT}$ for FSF and $\rho_{ABT}$ otherwise), and the bottom row to means over traits ($\bar{\rho_{A}}$ for FSF and $\bar{\rho_{AB}}$ otherwise). Parents of BPFs were sampled from ancestral population *Elite* and genotypes at SNP markers were used to calculate the genomic relationship matrix G. Results are shown for a random sample of 10,000 data points, $N_{train}=100$ and $h^{2}=0.6.$

Figure 3 shows scatter plots for empirical versus deterministic prediction accuracies for the standard scenario. In general, empirical and deterministic accuracies for single traits agreed relatively well for FSF ($r_{\rho ,\rho^{w}}=0.65$ and $r_{\rho ,\rho^{D}}=0.61$), but rather weakly for HSF (0.43 and 0.42, respectively) and URF (0.33 and 0.32, respectively). By comparison, the correlations between the means of empirical and deterministic accuracies across the 50 traits increased for FSF ($r_{\rho ,\rho^{w}}=0.81$ and $r_{\rho ,\rho^{D}}=0.65$), but even more so for HSF (0.94 and 0.92, respectively) and URF (0.89 and 0.88, respectively), indicating that trait-specific deviations from the mean empirical accuracy hampers the agreement with deterministic accuracies, particularly for HSF and URF.

For the general mean of empirical and deterministic PA across A,B, and T, $\bar{\rho^{W}}\;$matched very well with $\bar{\rho }$ for all pedigree relationships and values of $N_{train}$ and $h^{2}$ (Figure S2 in File S1). By comparison, $\bar{\rho^{D}}$ generally underestimated $\bar{\rho }$ with increasing bias for HSF and URF as compared with $\bar{\rho^{W}}$ (Figure S3 in File S1), and particularly for smaller values of $N_{train}$ and $h^{2}$ (Figure S2 in File S1). Calculating the G matrix from QTL instead of from SNP genotypes hardly influenced the bias of deterministic accuracies (Figure S4 in File S1) and the correlations with empirical accuracies.

### Causal analysis of the variation in PA among traits

**Figure 4:**
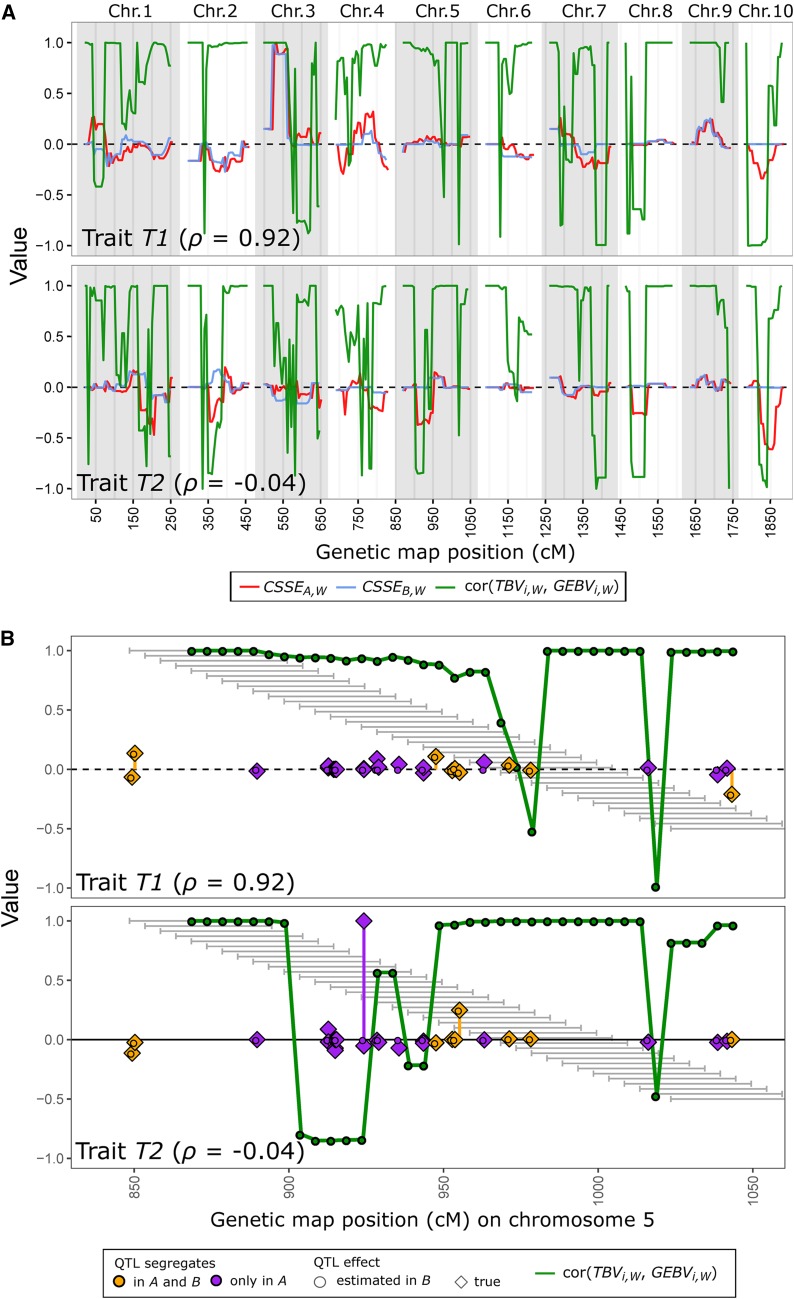
(A) Chromosome segment substitution effects (*CSSE_A_*,*_W_* in red and *CSSE_B_*,*_W_* in blue) and correlation between local TBVs and local GEBVs in the predicted family A (green) averaged in sliding windows W (see *Materials and Methods* for definition). GEBVs were calculated from QTL effects estimated by RR-BLUP in training set (HSF) B. Results are shown for $N_{train}=250$ and two traits *T1* and *T*2 with $h^{2}=1$ and large differences in prediction accuracy ρ. Both traits were generated from the same set of 1000 QTL with $\theta_{AB}\approx 0.40,$ but different QTL effects. (B) Correlation between local TBVs and local GEBVs (green lines) shown together with true QTL effects (diamonds) and estimated QTL effects (circles) for *T1* and *T*2 in B on chromosome 5. Colors indicate QTL segregating in both A and B (orange) or only in A (purple); grey bars in the background reflect the windows W.

Figure 4 compares two traits *T1* and *T2* with divergent PA for one representative pair of HSFs. For both traits with identical QTL positions and QTL genotypes in the BPF_pred_ A and BPF_train_ *B*, but different QTL effects, 376 QTL segregated in A, 286 in B and 151 of them jointly in A and B. For trait *T1* with high $\rho_{ABT}=0.92,$ the differences between chromosome segment substitution effects (*CSSE*) in A and B were generally small across the entire genome, in particular on chromosomes 2, 3, and 9, with sizeable *CSSEs* (Figure 4A). Conversely, for trait *T2* with low $\rho_{ABT}=-0.04,$ the *CSSEs* in A and B differed substantially over large parts of the genome, and showed even opposite signs on several chromosomes.

The correlation between local TBVs and local GEBVs of the DH lines k∈ A were closely associated with the differences between the *CSSEs* for A and B in the corresponding windows W (Figure 4A). If the difference in the *CSSE* for a segment was small, the correlation was generally high, particularly if both *CSSEs* in A and B had large magnitude and identical sign (see chromosomes 2, 3 and 9 for trait *T1*). Conversely, if the *CSSEs* for a window W differed and had opposite sign in A and B, the correlation between local TBV and local GEBV dropped substantially, and frequently became negative (see chromosomes 2, 5, and 8 for trait *T2*). Overall, the proportion of the genome showing low or even negative correlations was much smaller for trait *T1* with high PA than for trait *T2* with low PA.

Zooming into chromosome 5—which had a large impact on the differences between the two traits—revealed that for trait *T1*, all large-effect QTL that segregated in A also segregated in B (Figure 4B). However, for trait *T2*, there was a large-effect QTL that segregated only in A in windows W with low correlation between local TBVs and local GEBVs. Neighboring windows not harboring this QTL showed higher correlations. The trends for this exemplary chromosome were consistent with other chromosomes and other HSF pairs A and B, as well as other traits with high and low PA (results not shown).

## Discussion

Experimental studies showed that PA can be highly variable for GP within, but even more so across BPFs. Moreover, PA was found to vary substantially among different target traits for distinct pairs of training and predicted families. Investigating the causes for this variability is hardly possible based on experimental data due to the limited number and sample size of available BPFs, and the generally unknown genetic architecture of agronomically important traits. Here, we used computer simulations to analyze in detail why PA varies among different combinations of training sets, prediction sets, and polygenic traits. Moreover, we demonstrate that modification of available deterministic equations enables accurate estimates of PA averaged across many polygenic traits for both within-family GP and across-family GP.

### Variation in PA within and across biparental families

The average PA decreased under small $N_{train}$ and low $h^{2}$ (Figure 1A) for all pedigree relationships, as expected from theory (Daetwyler *et al.* 2008). This was always accompanied by a large increase in the variation of PA (Figure 1A), which was mainly caused by inflated residual errors [$\sigma_{\left(A:R\right)\times T}^{2}$ for FSF, $\sigma_{\left(A:B:R\right)\times T}^{2}$ for HSF and URF, Figure 1B]. These errors capture the variation in PA that arises due to the random sampling of (i) individuals (genotypes) from the BPF_train_, and (ii) their corresponding phenotypes for a specific trait. The larger residual errors in across-family GP are presumably due to incongruent sets of QTL segregating in pairs of HSFs and URFs, which can vary substantially across traits, as reflected by the SD of $\theta_{AB}$ (Table 2). The fact that predictions became much more robust under $N_{train}\geq$100 and $h^{2}\geq 0.6$ illustrate that large sample sizes and heritabilities are mandatory to alleviate the trait-specific sampling variance in PA. Together with the generally optimal conditions in within-family GP (Crossa *et al.* 2014), this nearly eliminated all variation in PA for FSF (Figure 1).

The predicted family BPF_pred_ accounted only for a marginal proportion of variation in PA, irrespective of the pedigree relationship with BPF_train_ (Figure 1B, $\sigma_{A}^{2}$). For within-family GP (where BPF_train_ = BPF_pred_), this implies that the genetic distance between the parents of a BPF has at best marginal influence on the average PA across traits, in agreement with previous studies (Lehermeier *et al.* 2014; Marulanda *et al.* 2015). This conclusion is further supported by the similar variation in PA among predicted families derived from the two ancestral populations ($\sigma_{A}^{2},$ Figure 2B, FSF), despite the much weaker latent pedigree structure in *Landrace* compared with *Elite* (Figure S1B in File S1). By comparison, the generally substantial influence of $\sigma_{A\times T}^{2}$ in FSF (Figure 1B and Figure 2B) suggests that PA strongly depends on $h^{2}$ in the training set (Figure S5 in File S1), which can be highly variable among BPF × trait combinations (Figure S6 in File S1). This is in harmony with previous studies that attributed variation in PA partially to differences in the phenotypic variance of the training set (Lehermeier *et al.* 2014; Marulanda *et al.* 2015).

For across-family GP, the expected PA depends largely on the pedigree relationship (Habier *et al.* 2007; Riedelsheimer *et al.* 2013) and on the variation in across-family genomic relationships. Since genomic relationships across families have a zero mean (if calculated according to Equation 1), their variation is equal to the mean squared genomic relationship between training and predicted individuals (Wientjes *et al.* 2013). Generally, PA is expected to increase proportionally with these squared relationships. In the case of BPFs, genomic relationships between families are heavily influenced by the proportion of polymorphic markers in the BPF_pred_ ($\theta_{AB}$) segregating also in the BPF_train_ (Figure S7 in File S1). Therefore, PA for across-family GP depends primarily on the magnitude of $\theta_{AB},$ because larger $\theta_{AB}$ implies that a greater proportion of the genetic variance in the BPF_pred_ can be explained by the QTL in BPF_train_. Accordingly, the variation in $\theta_{AB}$ among combinations of different HSFs or URFs (Figure S1D in File S1) was largely responsible for the notable contribution of $\sigma_{A:B}^{2}$ to the total variation in PA (Figure 1B). Altogether, the much larger $\sigma_{A:B}^{2}$ for across-family GP, compared to within-family GP, was mainly due to the overriding influence of $\sigma_{\left(A:B\right)\times T}^{2}$ besides the considerable contribution of $\sigma_{A:B}^{2}$ to $\sigma_{total}^{2}$ (Figure 1B, FSF *vs.* HSF or URF). Unraveling the genetic causes for this complex interaction required additional analyses, which are discussed in depth in the next section.

Sampling of training individuals from a given BPF_train_ barely contributed to the variation in PA, for both within- and across-family GP (Figure 1B, $\sigma_{A:R}^{2}$ and $\sigma_{A:B:R}^{2}$). Thus, compared with structured populations or diversity panels, there is little room for improvement by applying optimization algorithms accounting for genomic relationships in the sampling of training individuals within BPFs (Rincent *et al.* 2012; Akdemir *et al.* 2015; Bustos-Korts *et al.* 2016), confirming previous findings (Lorenz and Smith 2015; Marulanda *et al.* 2015). This is because already modest sample sizes (*e.g.*, *$N_{train}=50$*) enable the Mendelian sampling term in the BPF_train_ to be sufficiently captured. Nevertheless, we recommend $N_{train}\geq 50$ to achieve a high mean and small variance of PA (Table S3 in File S1) arising from sampling of genotypes from a given BPF_train_ (Figure 1B).

Previous experimental studies found generally higher levels of variation in PA, particularly for within-family GP (Riedelsheimer *et al.* 2013; Lehermeier *et al.* 2014; Lian *et al.* 2014). This is most likely attributable to miscellaneous additional factors present in these studies, which were not accounted for in our simulations. These factors include (i) small prediction set size, (ii) analysis of different types of progeny (F_2_ or backcross generations and DH lines derived from them), (iii) variation in QTL-SNP LD within BPFs due to low marker density, (iv) nonadditive gene action due to epistasis, and (v) estimation error in $h^{2},$ which affects calculation of PA from predictive ability. Further, the various agronomic traits investigated in the experimental studies differed likely in their genetic architecture, which further increases the total variation in PA compared with the polygenic traits simulated in our study ($\sigma_{T}^{2},$ Figure 1B). Consequently, our results should be regarded as a lower bound for the variation in PA that must be expected in practice for a given $N_{train}$ and $h^{2}.$

### Unraveling the variation among traits in across-family GP

We adopted the concept of local breeding values (*cf*. Kemper *et al.* 2015) to investigate the relationship between the strong variation in PA among traits and the large chromosome segments that DH lines of BPF inherit from their parents. The latter entails strong LD between QTL alleles and consequently small $M_{e}$ (Table 2), which is very different from the situation found in diverse populations such as cattle breeds (${\text{M}}_{\text{e}}\approx 1,000$) (Daetwyler *et al.* 2010; Wientjes *et al.* 2013). Thus, only a small number of local TBVs contribute to the “global” TBV of predicted individuals. Similarly, the PA can be thought of as the average accuracy of local GEBVs estimated from the training data, weighted by their relative contribution to the global TBV in the BPF_pred_. As a consequence of the small $M_{e}$ in BPFs, the accuracy of local GEBVs is prone to much larger sample variance than would be the case in more diverse populations. To illustrate this point, we examined for a given pair of HSFs exemplarily two traits with contrasting PA (Figure 4).

Of all QTL, only those that segregated in the BPF_pred_ (376/1, 000, Figure 4) contributed to the variance in local TBVs, which were estimated by local GEBVs from the training set. In our example, trait T1 with $\rho_{ABT}=0.92$ showed, on average, much higher correlations between local TBVs and local GEBVs in the BPF_pred_ along the entire genome than trait T2 with $\rho_{ABT}=-0.04$ (Figure 4A). For the trait with low PA, we found a larger proportion of local GEBVs that provided a false prediction signal, in the sense that negative effects were estimated for favorable parental chromosome segments and vice versa. These discrepancies between local TBVs and local GEBVs trace back to different chromosome segment substitution effects (*CSSE*, Equation 9) between the BPF_pred_ and BPF_train_ (Figure 4A), which, in the case of HSFs, occur if their noncommon parent carries different alleles at one or more QTL on the segment. If this is the case, one of the two BPFs will be monomorphic for the respective QTL. The effect of such a QTL compared with other QTL on a chromosome segment that may be polymorphic in both the BPF_pred_ and BPF_train_ determines the difference in *CSSE* between two families. For instance, if the variance in local TBVs among predicted individuals is dominated by a large-effect QTL, which is monomorphic in the training set, the ranking of local GEBVs based on the other polymorphic QTL located on this segment might deviate substantially from the ranking of local TBVs, resulting in low local PA (Figure 4B, T2). The frequency of inaccurate local GEBVs along the whole genome together with the variance explained by the corresponding local TBVs will finally determine the PA of across-family GP. Hence, two traits with the same number and positions of QTL might have very different PA, depending on the effects of QTL that are poly- or monomorphic across the training and prediction set. This explains also why $\theta_{AB},$ and thereby across-family genomic relationships, were closely associated with the average PA across many traits for different pairs of HSF and URF (Figure S7 in File S1), but poorly associated with PA $\rho_{ABT}$ for individual traits (Figure 3). Additional simulations showed further that reducing (i) the number of chromosomes on which QTL were located, or (ii) the total number of QTL, results in increased variation in PA (Figure S8 in File S1). Both these alterations reduce the number of local TBVs discernible for a trait, which underlines the relevance of small $M_{e}$ (*i.e.*, a low number of segments carrying QTL) for the variation in PA.

In conclusion, the large variation in PA among traits observed for across-family GP is caused by the strong LD among linked QTL within BPFs, and the resulting small effective number of chromosome segments contributing to polygenic traits, in combination with different QTL segregating across BPFs. Our analyses exemplify that BPFs represent a special case regarding the possibly strong fluctuations in PA, which is—to this extent—not expected for genetically more diverse populations.

### Influence of LD in the ancestral population on the expected accuracy of GP across BPFs

Differences in the extent of LD in ancestral populations *Elite* and *Landrace* (Figure S1A in File S1) translated into sizable differences in QTL-SNP linkage phase similarity among URFs derived from these populations (Figure S1C in File S1). Surprisingly, this barely affected $\bar{\rho }$ across URFs (Figure 2A and Table S3 in File S1). The low relevance of linkage phase similarity across URFs was confirmed by the similar PAs when substituting the SNP- with a QTL-derived G matrix (Figure 2A), which eliminates the influence of this factor. This reflects most likely the overriding influence of $\theta_{AB}$ on PA across URFs, because the mean $\theta_{AB}$ was similar for URFs derived from the two ancestral populations (Figure S1D in File S1). Thus, the higher mean in PA for HSFs compared with URFs seems to be attributable to higher $\theta_{AB}$ values (Table 2) rather than to the fact that QTL-SNP linkage phases are always consistent across HSF (Lehermeier *et al.* 2014), but not necessarily across URF. This corrects a conjecture of Riedelsheimer *et al.* (2013), who suspected that low PA obtained from certain URFs was due to low linkage phase similarity with the respective BPF_pred_.

### Deterministic equations for forecasting PA within and across BPFs

Forecasting PA based on estimated reliabilities of GEBVs requires that unrelated individuals have an expected genomic relationship of zero (Goddard *et al.* 2011; Wientjes *et al.* 2015). This can be achieved by a block-structured G matrix based on population-specific allele frequencies (*e.g.*, Chen *et al.* 2013). Preliminary analyses showed that in the calculation of G (Equation A5), correct treatment of SNPs polymorphic only in either BPF_train_ or in BPF_pred_ is very important. Different from empirical PAs, which remain unaffected by $\theta_{AB}<1$ (see Appendix A), deterministic PAs across BPFs can be grossly inflated by ignoring $\theta_{AB}<1$ in the calculation of G (results not shown). While $\theta_{AB}$ is generally high across diverse populations such as breeds of cattle (Matukumalli *et al.* 2009), it can fall to <0.4 across different BPFs produced from inbred parents in plant breeding (Figure S1D in File S1 and Table 2). Calculating G according to our improved method (Equation 1) largely eliminated the bias in deterministic accuracies $\rho^{W}$attributable to $\theta_{AB}<1$ and is therefore a prerequisite for applying Equation 3 to GP across BPFs.

Accounting for inbreeding (see Appendix B for derivation) in the original reliability equation, resulted together with the modifications on the G matrix in excellent agreement between empirical and deterministic accuracies $\rho^{W}$ averaged across traits, which is consistent with the findings of Wientjes *et al.* (2015) for cattle populations. However, the trait-dependent variation in empirical PA observed for GP across BPFs cannot be accounted for by $\rho^{W}.$ This is because for a given set of training and predicted individuals and two traits with the same $h^{2}$ but different QTL effects, the deterministic accuracy would be identical yet the empirical accuracy can differ substantially as illustrated in Figure 3 and Figure 4.

Forecasting PA within FSF by Daetwyler *et al.*’s (2008, 2010) equation based on population parameters has been widely used in plant breeding (Lorenz 2013; Riedelsheimer *et al.* 2013; Lian *et al.* 2014). However, estimates of $M_{e}$ can differ substantially (Riedelsheimer and Melchinger 2013; Wientjes *et al.* 2013) between the various proposed formulas to estimate $M_{e}$ from the effective population size $N_{e}$ and genome length (Goddard 2009; Meuwissen and Goddard 2010; Goddard *et al.* 2011). Moreover, estimation of $N_{e}$ itself is problematic, because it assumes a base population of unrelated founders, which is often impossible to define in practice (*cf*. Figure S1B in File S1, *Elite*). Following Goddard *et al.* (2011), we calculated $M_{e}$ directly from the variance of genomic relationships, with extensions devised by Wientjes *et al.* (2015, 2016) for GP across populations (Equation 5). This has the advantage that $M_{e}$ is computed from the actual genotypes for which the PA is to be forecasted. The calculation of $M_{e}$ required in Equation 4 must account for inbreeding (Equation 6), because the variance in genomic relationships increases with the inbreeding coefficient F (*see* Appendix C). Ignoring inbreeding would result in underestimation of $M_{e},$ and strong overestimation of the deterministic accuracy $\rho^{D}.$

An important assumption of the equation of Daetwyler *et al.* is that the entire genetic variance in the prediction set is explained by QTL segregating in the training set (*cf. $r_{\mathrm{Effect}}$* in Wientjes *et al.* 2016). This holds true for FSF ($\theta_{AB}=1$), but is violated for GP across BPFs ($\theta_{AB}\ll 1,$Table 2). As a solution for this problem, we propose multiplication with $\theta_{AB}$ in calculating $\rho^{D}$ (Equation 4), which efficiently reduced the strong upward-bias observed otherwise (results not shown). With these modifications, empirical and deterministic accuracies $\rho^{D}$ agreed reasonably well when averaged across traits, but forecasting was problematic for individual traits for the same reasons as discussed above for $\rho^{W}$ (Figure 3). Compared with previous experimental studies (Riedelsheimer *et al.* 2013; Lian *et al.* 2014), we found overall better agreement of ρ and $\rho^{D}$ for single traits in within-family GP (Figure 3). We suppose that, in addition to the lower variation in empirical PA (Figure 1), this is likely attributable to smaller deviations between estimated and true $M_{e}$ (Lian *et al.* 2014) when dealing with real traits of diverse genetic architecture.

An upward bias in deterministic PA must generally be expected if SNPs are not a good approximation of QTL due to incomplete QTL-SNP LD, (*cf. $r_{\mathrm{Effect}}$* *vs.* ${\text{r}}_{\text{LD}}$ in Wientjes *et al.* 2016), leading to “missing heritability” in genomic studies (Yang *et al.* 2010). This is because empirical PA decreases as less variance at QTL is explained by SNPs under incomplete LD, whereas deterministic PA is hardly affected (Figure S9 in File S1). However, our results show that this is barely relevant in BPFs (Figure 3 *vs.* Figure S4 in File S1), if large chromosome segments are covered sufficiently by markers. Thus, a sizable reduction in empirical PA and overestimation of deterministic PA must only be expected under very low marker density (<100 SNPs) as in the study of Lian *et al.* (2014). Although these authors argued that 100 SNPs were likely sufficient for within-family GP in maize, our results indicate that at least 1000 and 2500 SNPs should be used for within- and across-family GP, respectively, to obtain acceptable empirical PA and minimize the bias in deterministic PA (Figure S9 in File S1). If such numbers are not available, deterministic equations must additionally account for incomplete LD (Wientjes *et al.* 2016), using, for example, multiplication with the average LD ($r^{2}$) between adjacent markers as proxy for the QTL-SNP LD (Lian *et al.* 2014).

Besides low marker density, incomplete QTL-SNP LD can result from differences in the allele frequency distribution at QTL and SNPs (Goddard *et al.* 2011), *inter alia* due to ascertainment bias of SNP chips. These differences are in reality unknown, and, as treated herein, commonly not accounted for in simulation studies (Daetwyler *et al.* 2013). For GP across BPFs, differences in allele frequencies at QTL and SNPs in the ancestral population (*cf*. Figure S1E in File S1) would translate into different $\theta_{AB}$ values at SNPs and QTL across BPFs, because the smaller the minor allele frequency, the larger the chances of a locus being monomorphic in a BPF. Thus, calculation of $\rho^{D}$ might be inflated by an upward-bias in $\theta_{AB}$ (Equation 5), in addition to the possible overestimation of across-family genomic relationships affecting both $\rho^{D}$ and $\rho^{W}$ (Equations 3 and 4). Further research is needed to show how strongly overestimation of $\theta_{AB}$ can affect application of deterministic equations in practice, for example, by comparing the equations under chip-based and sequencing-based genotyping (Pérez-Enciso *et al.* 2015).

We assumed in our derivations that the genetic correlation among BPFs = 1 (*see* Appendix B), which is expected to hold under a purely additive-genetic model, as applies in the absence of epistasis to (i) testcross performance for a given tester, and (ii) to *per se* performance of completely homozygous lines (Melchinger 1987). By comparison, in cattle breeds or diverse germplasm in plant breeding, genetic correlations between populations are typically < 1 (Karoui *et al.* 2012; Lehermeier *et al.* 2015). Accounting for genetic correlations is possible with multi-group models, but these require sufficient phenotypic data for the predicted population as well as estimating these correlations, which seems impractical in the case of GP of a single BPF.

Despite generally promising results for both deterministic equations, we recommend using $\rho^{W}$ (Equation 3), because it depended less on the relatedness between BPFs, $N_{train},$ and $h^{2}$ (Figures S2 and S3 in File S1), rendering it more robust across a wide range of scenarios. Since $\rho^{W}$ and $\rho^{D}$ (as implemented here) require genotypic data of both the training and predicted individuals, they can be applied only after obtaining genotypic data of the individuals to be predicted. Alternatively, for newly planned crosses we propose to use computer simulations to generate *in silico* virtual genotypic data of the corresponding BPFs using known genotypes of the parents and genetic map information of the markers, as conducted in this study (*cf*. Mohammadi *et al.* 2015). This would make both equations accessible prior to generating new crosses for use in optimizing training set designs and allocation of resources.

### Conclusions and extensions to multi-family training sets

We demonstrated that the empirical PA in BPFs of inbred lines is prone to various sources of variation, which differ strongly in their relevance for GP within and across BPFs. It should be stressed that the conclusions drawn from our study do not only apply to DH lines, but also to inbreds developed by recurrent selfing and most likely also to partly inbred generations. Overall, our results corroborate within-family GP as a valuable and robust tool for the implementation of GP in plant breeding, provided the training set meets minimum standards for $N_{train}$ (≥50) and $h^{2}$ (>0.3). However, the need for phenotypes from the predicted family represents the main drawback of within-family GP, because this increases both the costs and the time needed until selection can be applied.

Our simulations on across-family GP were restricted to the simple strategy of using only a single HSF or URF for model training. This provided a manageable framework for analyzing the underlying causes affecting variation in PA. For a given BPF_pred_, we showed: (i) the PA in across-family GP expected across many traits differs systematically between different BPF_train_, even if they have the same pedigree relationship with the BPF_pred_, (ii) deterministic equations enable accurate forecasts of the PA across traits for given pairs of BPF_pred_ and BPF_train_, and (iii) large variation in the PA among traits hampers the forecasting. Therefore, it is very unlikely to find a single BPF_train_ that performs uniformly best across all target traits. This means that caution must be exercised when applying rules of thumb or deterministic equations for choosing the BPF_train_ in GP of a specific trait given BPF_pred_. This issue can be even more severe if (i) traits deviate from the polygenic architecture assumed in our simulations, or (ii) $M_{e}$ in the BPFs is smaller than $M_{e}$ in maize due to fewer chromosomes and/or smaller genome size (Figure S8 in File S1). Thus, identification of useful, trait-specific BPF_train_ might only be possible by directly evaluating the empirical PA for a small sample (N∼30) of individuals from the BPF_pred_. However, this would largely eliminate the time- and cost-related advantages of genomic selection based on previously available data from BPFs.

In practice, breeders generally do not rely on single-family training sets in GP across BPFs, but rather use multi-family training set designs for the sake of increasing sample size (Heffner *et al.* 2011; Riedelsheimer *et al.* 2013; Hickey *et al.* 2014; Jacobson *et al.* 2014; Lehermeier *et al.* 2014). Another important advantage of multi-family over single-family training sets in across-family GP most likely stems from the increased proportion of causal loci segregating in both the BPF_pred_ and the training set, which we identified as the core problem leading to the large variation of PA in GP across single BPFs. One critical question in this context is whether or not a single BPF_train_ that is poorly predictive of a given BPF_pred_ (*e.g.*, a HSFs that yields PA close to zero, Figure 4) is detrimental or harmless for PA if combined together with other predictive BPFs for extending the training set. The problem might exacerbate if URF are included in multi-family training sets (*cf*. Albrecht *et al.* 2011), which might come at the expense of reduced linkage phase similarity (*cf*. Figure S1C in File S1) between a multifamily training set and the BPF_pred_ (Lorenz and Smith 2015). Further research is warranted to investigate whether the current design of training sets can be improved by identifying and excluding adverse families to avoid disappointing outcomes of GP in BPFs.

## Supplementary Material

Supplemental material is available online at www.g3journal.org/lookup/suppl/doi:10.1534/g3.117.300076/-/DC1.

## Appendix A

### Genomic Relationships Between DH Lines from BFPs A and B Calculated with Different Methods

Suppose i∈A and j∈B are two DH lines from BPFs A and B, respectively. Let $L=L_{A\cup B},L_{A},$ $L_{B}$ and $L_{A\cap B}$ denote the set of loci (SNPs or QTL, depending on the context) that are polymorphic in A or in B, polymorphic in A, polymorphic in B, and polymorphic in A and B, respectively. Since A and B are BPFs, we have, under Mendelian inheritance,

$$p_{A,k}=\left\{\begin{array}{c} \frac{1}{2}\text{for}\;k\in L_{A}\\ 0\;\text{or}\;1\;\text{elsewhere} \end{array}\right\}\;\text{and}\;p_{B,k}=\left\{\begin{array}{c} \frac{1}{2}\text{for}\;k\in L_{B}\\ 0\;\text{or}\;1\;\text{elsewhere} \end{array}\right\}.$$ (A1)

Thus,

$$\sum_{k\in L}2p_{A,k}\left(1-p_{A,k}\right)=\frac{1}{2}\left|L_{A}\right|\;\text{and}\;\sum_{k\in L}2p_{B,k}\left(1-p_{B,k}\right)=\frac{1}{2}\left|L_{B}\right|,$$ (A2)

where $\left|L_{A}\right|$ and $\left|L_{B}\right|$ denote the number of elements in set $L_{A}$ and $L_{B},$ respectively. Defining $w_{A_{i},k}=\left(x_{A_{i},k}-2p_{A,k}\right),$ $w_{B_{j},k}=\left(x_{B_{j},k}-2p_{B,k}\right)$ and $z_{A_{i}B_{j},k}=w_{A_{i},k}w_{B_{j},k},$ we get, with Equation 1, for completely homozygous lines

$$z_{A_{i}B_{j},k}=\left\{\begin{array}{c} +1\;\text{if}\;k\in L_{A\cap B}\;\text{and}\;x_{A_{i},k}=x_{B_{j},k}\;\left(i.e.,\text{identical}\;\text{alleles}\;\text{in}\;i\;\text{and}\;j\;\text{at}\;\text{locus}\;k\right)\\ -1\;\text{if}\;k\in L_{A\cap B}\;\text{and}\;x_{A_{i},k}\neq x_{B_{j},k}\;\left(i.e.,\text{different}\;\text{alleles}\;\text{in}\;i\;\text{and}\;j\;\text{at}\;\text{locus}\;k\right)\\ 0\;\text{elsewhere} \end{array}\right\}.\;$$ (A3)

For calculating the elements of the genomic relationship matrix $G_{AB}=\left(G_{A_{i}B_{j}}\right)$ according to the modification proposed in Equation 1, we obtain

$$G_{A_{i}B_{j}}=\frac{\sum_{k\in L}z_{A_{i}B_{j},k}}{\sqrt{\frac{1}{2}\left|L_{A}\right|}\sqrt{\frac{1}{2}\left|L_{B}\right|}}=\frac{2\left|L_{A\cap B}\right|\left[2SM_{i,j}\left(L_{A\cap B}\right)-1\right]}{\sqrt{\left|L_{A}\right|\left|L_{B}\right|}},$$ (A4)

where $SM_{i,j}\left(L_{A\cap B}\right)$ refers to the simple matching coefficient (Sneath and Sokal 1973), also known as the IBS (identity by state) coefficient (Astle and Balding 2009), between i and j with respect to the loci set $L_{A\cap B}.$ Using the original formula of Chen *et al.* (2013), which extends Method 1 of VanRaden (2008) to the case of two populations, we obtain the genomic relationship matrix $G_{AB}^{VR1}=\left(G_{A_{i}B_{j}}^{VR1}\right),$ with elements

$$G_{A_{i}B_{j}}^{VR1}=\frac{\sum_{k\in L}z_{A_{i}B_{j},k}}{2\sum_{k\in L}\sqrt{p_{A,k}\left(1-p_{A,k}\right)p_{B,k}\left(1-p_{B,k}\right)}}=\frac{\left|L_{A\cap B}\right|\left[2SM_{i,j}\left(L_{A\cap B}\right)-1\right]}{2\left|L_{A\cap B}\right|\sqrt{\frac{1}{16}}}.\;=2\left[2SM_{i,j}\left(L_{A\cap B}\right)-1\right]\;$$ (A5)

Extending Method 2 of VanRaden (2008) to the case of two populations, we obtain the genomic relationship matrix $G_{AB}^{VR2}=\left(G_{A_{i}B_{j}}^{VR2}\right),$ as follows

$$G_{A_{i}B_{j}}^{VR2}=\frac{1}{\left|L_{A\cap B}\right|}\sum_{k\in L_{A\cap B}}\frac{z_{A_{i}B_{j},k}}{\sqrt{2p_{A,k}\left(1-p_{A,k}\right)p_{B,k}\left(1-p_{B,k}\right)}}=\frac{2}{\left|L_{A\cap B}\right|}\sum_{k\in L_{A\cap B}}z_{A_{i}B_{j},k}\;\;=2\left[2SM_{i,j}\left(L_{A\cap B}\right)-1\right],$$ (A6)

where summation is only possible for $k\in L_{A\cap B},$ because for $k\in \;L_{A^{c}\cup B^{c}}$ the denominator is zero, where $L_{A^{c}\cup B^{c}}$ denotes subset of L polymorphic in B but not in A, or polymorphic in A but not in B. Thus, we obtain

$$G_{A_{i}B_{j}}^{VR1}=G_{A_{i}B_{j}}^{VR2}=2\left[2SM_{i,j}\left(L_{A\cap B}\right)-1\right]=\gamma G_{A_{i}B_{j}},$$ (A7)

in BPFs with allele frequencies equal to 0.5 at segregating loci, with $\gamma =\sqrt{\left|L_{A}\right|\left|L_{B}\right|}/\left|L_{A\cap B}\right|.$ Consequently, γ=1 if and only if $L_{A}=L_{B}$ (*e.g.*, if A=B), but otherwise γ>1. Note that the empirical PA of GBLUP is invariant to γ (*cf*. Strandén and Christensen 2011), but γ affects uniformly the scaling of GEBVs and reliabilities thereof. Note also that $SM_{i,j}$ calculated with regard to all loci (set L) can deviate from $SM_{i,j}\left(L_{A\cap B}\right),$ because

$$SM_{i,j}=\frac{\left|L_{A\cap B}\right|}{\left|L\right|}SM_{i,j}\left(L_{A\cap B}\right)+\frac{\left|L_{A\cap B^{c}}\right|}{\left|L\right|}SM_{i,j}\left(L_{A\cap B^{c}}\right)+\frac{\left|L_{A^{c}\cap B}\right|}{\left|L\right|}SM_{i,j}\left(L_{A^{c}\cap B}\right),$$ (A8)

and $SM_{i,j}\left(L_{A\cap B^{c}}\right)$ as well as $SM_{i,j}\left(L_{B\cap A^{c}}\right)$ can vary between pairs of i and j, where $L_{A\cap B^{c}}$ and $L_{A^{c}\cap B}$ denote subsets of L polymorphic in A but not in B, and polymorphic in B but not in A, respectively.

## Appendix B

### Calculation of the Reliability of GP across Populations for Inbred Individuals

Assume two populations *A* (= prediction set) and *B* (= training set), which are not necessarily BPFs, and we consider across-population GP. Using well-known results about selection indices (Mrode 2005), the breeding value $g_{A_{i}}$ for individual i∈A, which may be inbred, is predicted with information from its genotype and the phenotypic and genotypic information from the training set as:

$${\hat{g}}_{A_{i}}=\text{cov}(g_{A_{i}},y_{B}){\left[\text{var}(y_{B})\right]}^{-1}y_{B},$$ (B1)

in which ${\hat{g}}_{A_{i}}$ is the predicted breeding value, $g_{A_{i}}$ is the true breeding value of individual i in population A, and $y_{B}$ is a vector with phenotypes of individuals from population B, corrected for fixed effects.

The covariance between the true breeding value of an individual from population A and the phenotypes of individuals from population B is:

$$\text{cov}\left(g_{A_{i}},y_{B}\right)=\text{cov}\left(g_{A_{i}},g_{B}+e_{B}\right)=\text{cov}\left(g_{A_{i}},g_{B}\right)+\text{cov}\left(g_{A_{i}},e_{B}\right)=G_{A_{i},B}^{T}r_{AB}\sigma_{u_{A}}\sigma_{u_{B}},$$ (B2)

where $r_{AB}$ is the genetic correlation between A and B (which represents the correlation between the breeding value in population A and the breeding value in population B for the individuals in A), $G_{A_{i},B}$ is the vector of genomic relationships between individual i and the training individuals from B that can be estimated by Equation 1 in the main text, and $\sigma_{u_{A}}$ and $\sigma_{u_{B}}$ are the square root of the additive variances in A and B, respectively. Finally,

$$\mathrm{var}\left(y_{B}\right)=\mathrm{cov}\left(y_{B},y_{B}\right)=\mathrm{cov}\left(g_{B}+e_{B},g_{B}+e_{B}\right)=\mathrm{cov}\left(g_{B},g_{B}\right)+\mathrm{cov}\left(e_{B},e_{B}\right)=G_{BB}\sigma_{u_{B}}^{2}+R_{B}\sigma_{e_{B}}^{2},$$ (B3)

where $G_{BB}$ is the genomic relationship matrix among training individuals in B, $R_{B}\sigma_{e_{B}}^{2}$ is the covariance matrix of the errors $e_{B}$ in the observation vector $y_{B},$ and the breeding value $u_{A_{i}}$ is predicted as:

$${\hat{g}}_{A_{i}}=G_{A_{i},B}^{T}r_{AB}\sigma_{u_{A}}\sigma_{u_{B}}{\left[G_{BB}\sigma_{u_{B}}^{2}+R_{B}\sigma_{e_{B}}^{2}\right]}^{-1}y_{B}=r_{AB}G_{A_{i},B}^{T}\frac{\sigma_{u_{A}}}{\sigma_{u_{B}}}{\left[G_{BB}+R_{B}\frac{\sigma_{e_{B}}^{2}}{\sigma_{u_{B}}^{2}}\right]}^{-1}y_{B}.$$ (B4)

We are interested in the reliability

$$r_{A_{i}}^{2}=\frac{{\text{Cov}}^{2}\left({\hat{g}}_{A_{i}},g_{A_{i}}\right)}{\sigma_{{\hat{g}}_{A}}^{2}\sigma_{g_{A}}^{2}}=\frac{\text{Cov}\left({\hat{g}}_{A_{i}},g_{A_{i}}\right)}{\sigma_{g_{A}}^{2}}\;$$ (B5)

for the estimated breeding value ${\hat{g}}_{A_{i}}$ of individual i∈A, $g_{A_{i}}$ being defined with respect to population A. Since $\mathrm{var}\left(g_{A_{i}}\right)=\sigma_{g_{A}}^{2}=G_{A_{i},A_{i}}\sigma_{u_{A}}^{2},$ together with Equation B2, we obtain

$$r_{A_{i}}^{2}=\frac{\text{Cov}\left({\hat{g}}_{A_{i}},g_{A_{i}}\right)}{\sigma_{g_{A}}^{2}}=\frac{\text{Cov}\left({\hat{g}}_{A_{i}},g_{A_{i}}\right)}{G_{A_{i},A_{i}}\sigma_{u_{A}}^{2}}=\text{cov}\left(\frac{r_{AB}G_{A_{i},B}^{T}\frac{\sigma_{u_{A}}}{\sigma_{u_{B}}}{\left[G_{BB}+R_{B}\frac{\sigma_{e_{B}}^{2}}{\sigma_{u_{B}}^{2}}\right]}^{-1}y_{B},u_{A_{i}}\;}{G_{A_{i},A_{i}}\sigma_{u_{A}}^{2}}\right)=\frac{r_{AB}G_{A_{i},B}^{T}\frac{\sigma_{u_{A}}}{\sigma_{u_{B}}}{\left[G_{BB}+R_{B}\frac{\sigma_{e_{B}}^{2}}{\sigma_{u_{B}}^{2}}\right]}^{-1}}{G_{A_{i},A_{i}}\sigma_{u_{A}}^{2}}\mathrm{cov}\left(y_{B},u_{A_{i}}\right)=\frac{r_{AB}G_{A_{i},B}^{T}\frac{\sigma_{u_{A}}}{\sigma_{u_{B}}}{\left[G_{BB}+R_{B}\frac{\sigma_{e_{B}}^{2}}{\sigma_{u_{B}}^{2}}\right]}^{-1}r_{AB}G_{A_{i},B}\sigma_{u_{A}}\sigma_{u_{B}}}{G_{A_{i},A_{i}}\sigma_{u_{A}}^{2}}$$ (B6)

so, the reliability is:

$$r_{A_{i}}^{2}=r_{AB}^{2}\frac{G_{A_{i},B}^{T}{\left[G_{BB}+R_{B}\frac{\sigma_{e_{B}}^{2}}{\sigma_{u_{B}}^{2}}\right]}^{-1}G_{A_{i},B}}{G_{A_{i},A_{i}}}.$$

## Appendix C

### Calculation of $M_{e}$ and the Variance of Genomic Relationships of Inbred Populations

Consider two populations A (=prediction set) and B (= training set) that are not necessarily BPFs. Based on the theory of Goddard *et al.* (2011), Wientjes *et al.* (2015) suggested to calculate the effective number of chromosome segments $M_{e_{A,B}}$ shared between the two populations as (see their Equation 20)

$$M_{e_{A,B}}=\frac{1}{\mathrm{var}\left(G_{A_{i}B_{i}}-\text{E}\left(G_{A_{i}B_{i}}\right)\right)}$$ (C1)

If all individuals in A have the same pedigree relationships with the individuals in B, which holds true for pairs of BPFs, we have $\text{E}\left(G_{A_{i}B_{i}}\right)=\text{constant}\;\forall i\in A\;\text{and }\forall i\in B$ so that $\mathrm{var}\left(G_{A_{i}B_{i}}-\text{E}\left(G_{A_{i}B_{i}}\right)\right)=\mathrm{var}\left(G_{A_{i}B_{i}}\right).$ If the genotypes of loci pairs are stochastically independent, as follows from the assumption of independent segregation in the definition of $M_{e}$ (Daetwyler *et al.* 2010), and applies in reality to all loci pairs located on different chromosomes (*i.e.*, a fraction of at least (C−1)/C of all loci pairs, assuming C chromosomes with equal length and number of loci), we have

$$\mathrm{var}\left(G_{A_{i}B_{i}}\right)=\frac{\mathrm{var}\left(\sum_{k\in L}w_{A_{i},k}w_{B_{j},k}\right)}{\sum_{k\in L}2p_{A,k}\left(1-p_{A,k}\right)\sum_{k\in L}2p_{B,k}\left(1-p_{B,k}\right)}=\frac{\sum_{k\in L}\mathrm{var}\left(w_{A_{i},k}w_{B_{j},k}\right)}{\sum_{k\in L}2p_{A,k}\left(1-p_{A,k}\right)\sum_{k\in L}2p_{B,k}\left(1-p_{B,k}\right)},$$ (C2)

where $w_{A_{i},k}$ and $w_{B_{j},k}$ are defined as in Appendix A, with $\text{E}\left(w_{A_{i},k}\right)=0$ and $\text{E}\left(w_{B_{j},k}\right)=0$ and $w_{A_{i},k}$ and $w_{B_{j},k}\;$are stochastically independent because i and j are random samples from A and B, respectively. Thus, using results about the product of two stochastically independent random variables (Mood 1974), we obtain

$$\mathrm{var}\left(w_{A_{i},k}w_{B_{j},k}\right)=\mathrm{var}\left(w_{A_{i},k}\right)\mathrm{var}\left(w_{B_{j},k}\right).$$ (C3)

Under inbreeding with inbreeding coefficient $F_{A}$ in population A, applying well-known results on the effects of inbreeding on the additive genetic variance (Falconer and Mackay 1996), we obtain

$$\mathrm{var}\left(w_{A_{i},k}|i\;\text{with}\;F_{A}\right)=\left(1+F_{A}\right)\mathrm{var}\left(w_{A_{s},k}|s\;\text{with}\;F_{s}=0\right)$$ (C4)

and a similar result for population B. Combining Equations C2, C3, and C4 yields

$$\mathrm{var}\left(G_{A_{i},B_{j}}|i\;\text{with}\;F_{A}\wedge j\;\text{with}\;F_{B}\right)=\;\left(1+F_{A}\right)\left(1+F_{B}\right)\mathrm{var}\left(G_{A_{s},B_{t}}|s\;\text{with}\;F_{s}=0\wedge t\;\text{with}\;F_{t}=0\right)$$ (C5)

While, for inbred generations derived by recurrent selfing, this equation may hold only approximately true due to the assumption of stochastic independence among all loci pairs, a proof can be given that Equation C5 holds strictly true without this requirement for DH lines and F_2_ individuals, *i.e.*,

$$\mathrm{var}\left(G_{A_{i},B_{j}}|i=\text{DH}\;\text{line}\wedge j=\text{DH}\;\text{line}\right)=4\mathrm{var}\left(G_{A_{s},B_{t}}|s={\text{F}}_{2}\;\text{ind}.\wedge s={\text{F}}_{2}\;\text{ind}.\right)$$ (C6)

In the original publications (Goddard *et al.* 2011; Wientjes *et al.* 2015) connecting $M_{e}$ with the variance in genomic relationships among individuals, it was assumed that the individuals were noninbred. However, if they are DH lines or from another inbred generation, this is expected to affect $M_{e},$ so that for the case of fixed pedigree relationships between A and B, the estimator of $M_{e}$ becomes

$$M_{e_{A,B}}=\frac{\left(1+F_{A}\right)\left(1+F_{B}\right)}{\mathrm{var}\left(G_{A_{i}B_{i}}|i\;\text{with }\;F_{A}\wedge j\;\text{with}\;F_{B}\right)}.$$ (C7)
