# Supplementary material for: Genomic Prediction Within and Across Biparental Families: Means and Variances of Prediction Accuracy and Usefulness of Deterministic Equations
Source: G3 (Bethesda). 2017 Sep 15;7(11):3571–86. doi: 10.1534/g3.117.300076 (PMC5677162; doi:10.1534/g3.117.300076)
Supplement: Supplementary file 1 [file 3571FileS1.pdf]

Figures – Supplemental Information

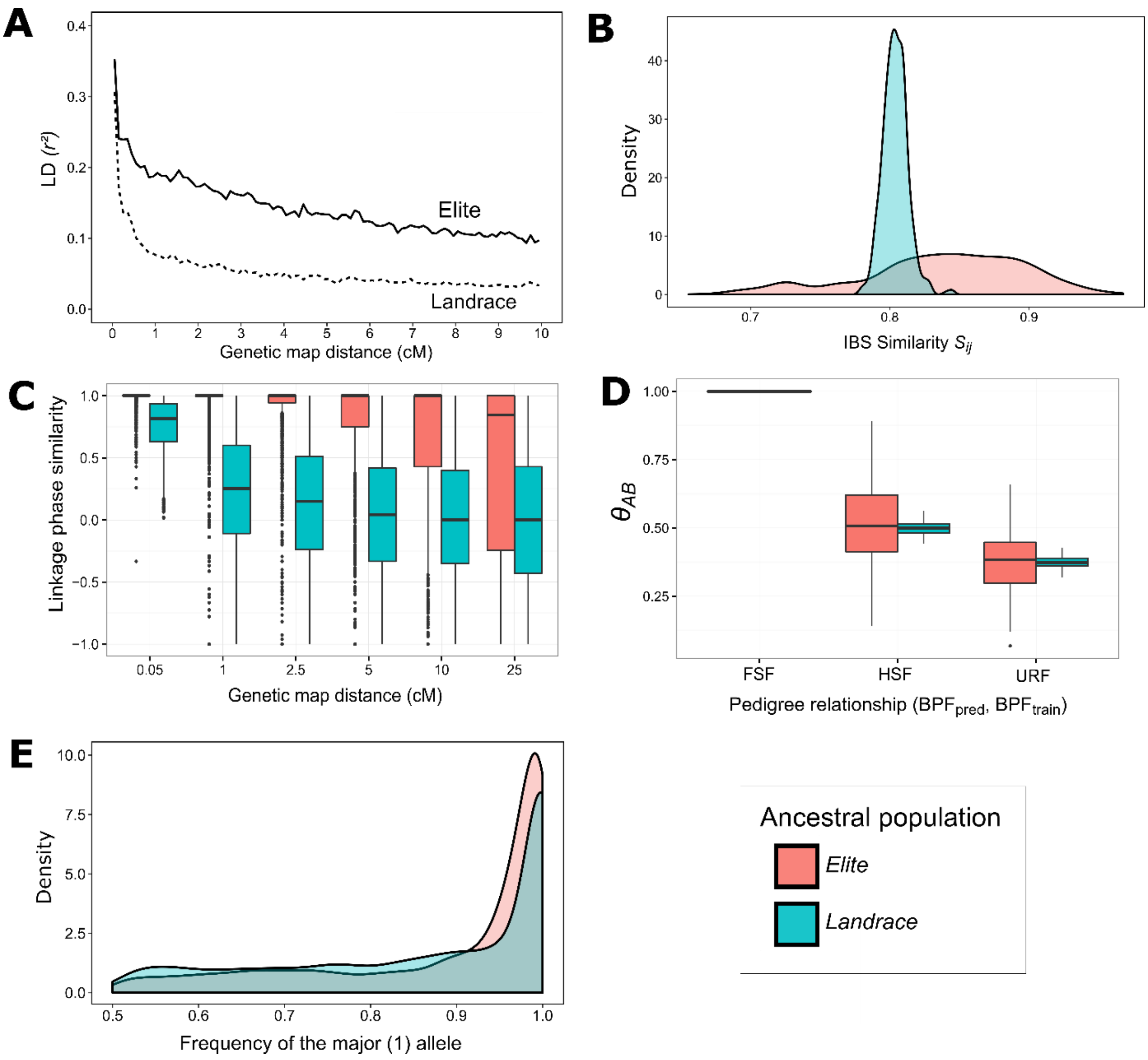

Figure S1 Comparison of the two ancestral populations *Elite* and *Landrace* from which parents of biparental families of doubled-haploid (DH) lines were sampled. **(A)** Linkage disequilibrium (LD) between loci pairs measured as r^2 plotted against their genetic map distance in cM. **(B)** Distribution of pairwise genetic similarity coefficients S_{ij} among individuals i and j. **(C)** **D**istribution of linkage phase similarity values between two pairs of unrelated biparental families (BPF), measured as cosine similarity between r values of pairs of loci that are polymorphic in both sets of parents (Schopp *et al.* 2017), for different intervals of genetic map distance ($\pm$ 0.1 cM). **(D)** Distribution of θ_{AB} for different pairs of predicted families (BPF$_{pred}$) A and training families (BPF$_{train}$) B derived from the two ancestral populations, calculated as the number of loci that segregate in both A and B, divided by the number of loci that segregate in A, for different pedigree relationships (full-sib, FSF; half-sib, HSF; unrelated family, URF) between A and B. **(E)** Distribution of major allele frequency for all loci in the ancestral population.

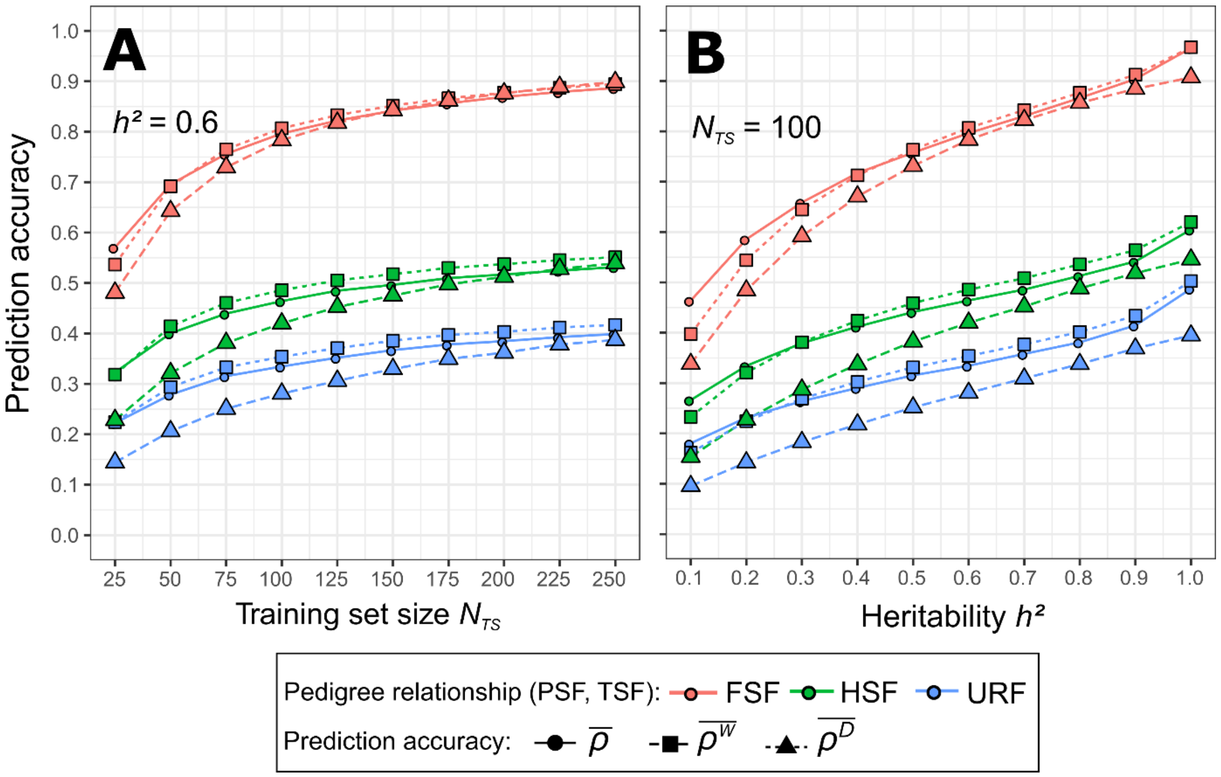

Figure S2 Comparison of mean values (averaged across traits and BPFs) of empirical prediction accuracy $\bar{\rho}$ and deterministic prediction accuracies $\overline{\rho^W}$ and $\overline{\rho^D}$ in biparental families (BPFs) of doubled-haploid lines for **(A)** different sample sizes N_{train} assuming $h^2 = 0.6$ and **(B)** different h^2 assuming $N_{train} = 100$. Parents of BPFs were sampled from ancestral population *Elite* and genotypes at SNP markers were used to calculate the genomic relationship matrix $\boldsymbol{G}$. Results are shown for the three pedigree relationships (full-sib, FSF; half-sib, HSF; unrelated family, URF) between the predicted family (BPF_{pred}) A and the training family ($\text{BPF}_{\text{train}}$) B.

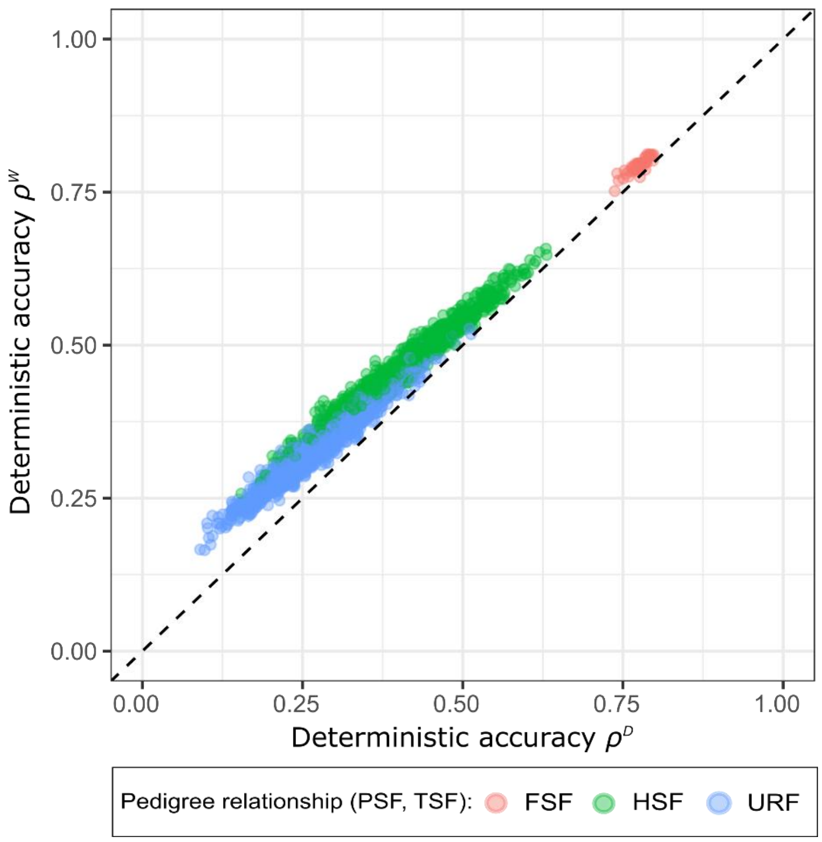

Figure S3 Deterministic prediction accuracy ρ^D plotted against deterministic prediction accuracy ρ^W in biparental families (BPFs) of doubled-haploid lines. Results refer to means across traits. Parents of BPFs were sampled from ancestral population *Elite* and genotypes at SNP markers were used to calculate the genomic relationship matrix $\boldsymbol{G}$. Results are shown for a random sample of 10,000 data points, $N_{train} = 100$ and $h^2 = 0.6$.

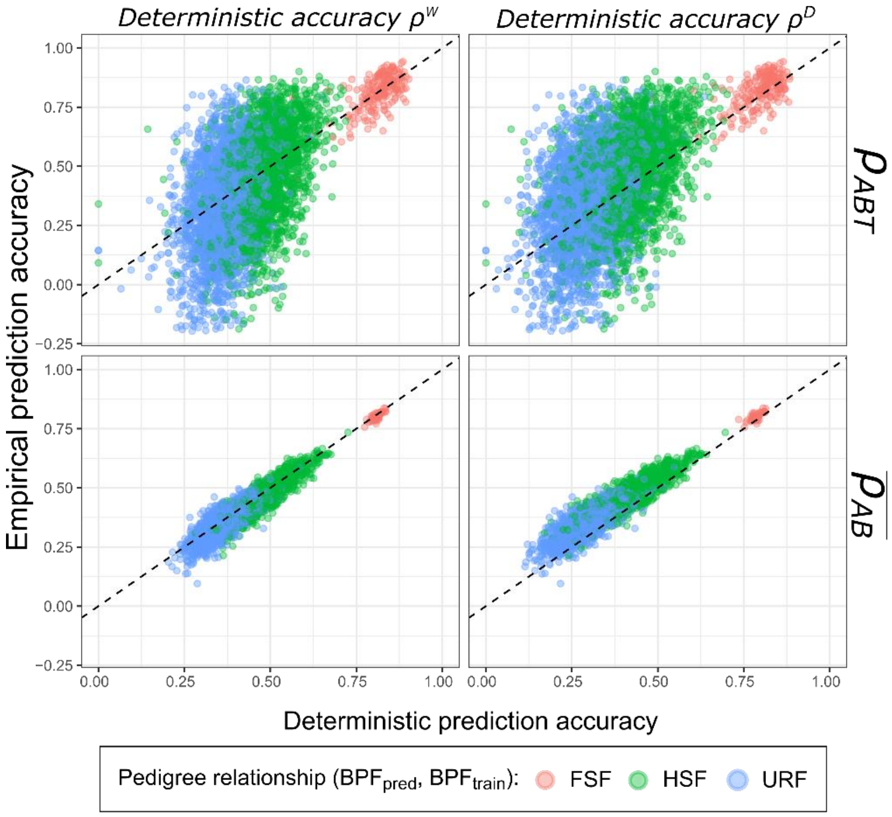

Figure S4 Empirical prediction accuracy ρ in biparental families (BPFs) of doubled-haploid lines plotted against deterministic prediction accuracies ρ^W and ρ^D. The top two graphs refer to observations for single traits (ρ_{AT} for FSF and ρ_{ABT} otherwise) and the bottom row to means over traits ($\overline{\rho_A}$ for FSF and $\overline{\rho_{AB}}$ otherwise). Parents of BPFs were sampled from ancestral population *Elite* and genotypes at QTL were used to calculate the genomic relationship matrix $\boldsymbol{G}$. Results are shown for a random sample of 10,000 data points, $N_{train} = 100$ and $h^2 = 0.6$.

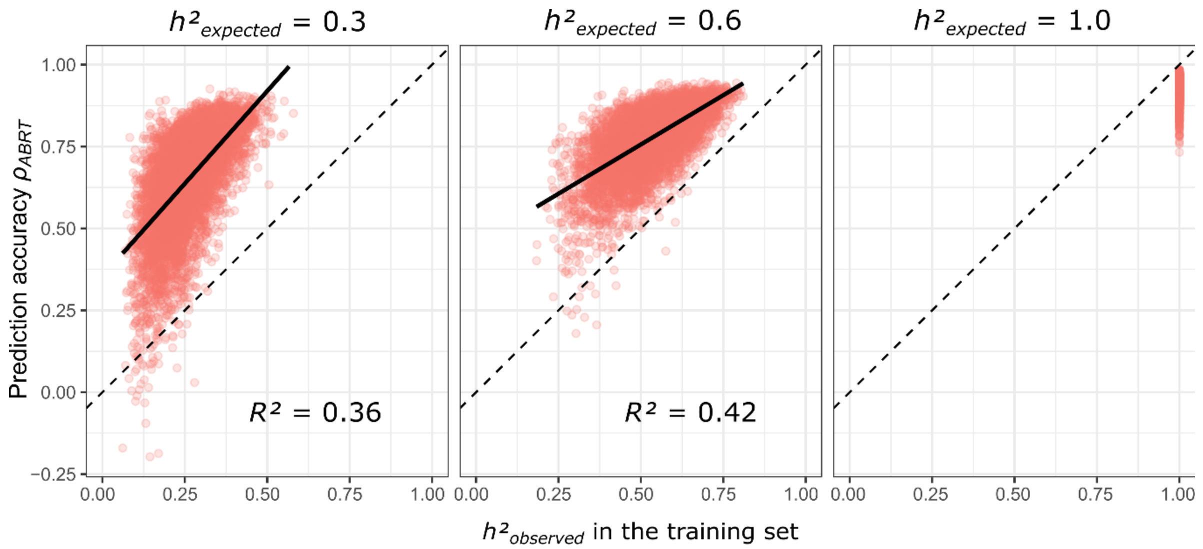

Figure S5 Empirical prediction accuracy ρ_{ABT} within biparental families (BPFs) of doubled-haploid lines assuming model training was performed in the same BPF, plotted against h^2_{observed} in the training set. Results are shown for different levels of h^2_{expected} defined according to the average genetic variance across all simulated BPFs and traits (*see Material and Methods* for details). Parents of BPFs were sampled from ancestral population *Elite* and genotypes at SNP markers were used to calculate the genomic relationship matrix $\boldsymbol{G}$. Results refer to $N_{train} = 100$.

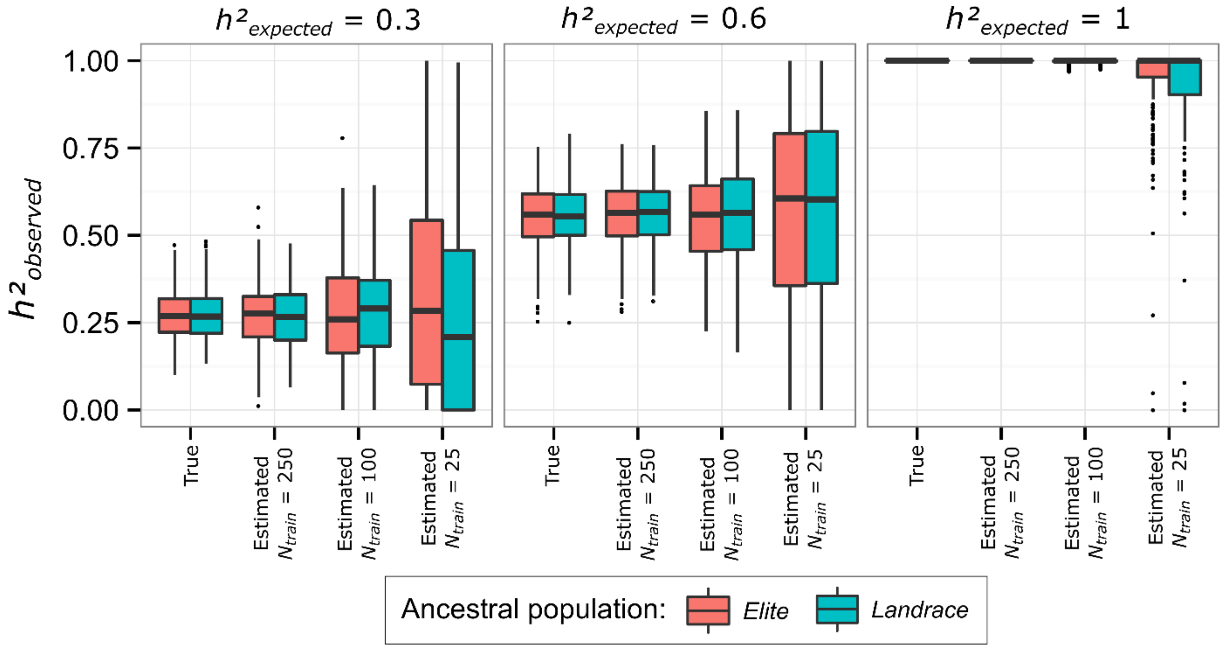

Figure S6 Boxplots of h^2_{observed} within biparental families (BPFs) of doubled-haploid lines for different levels of h^2_{expected}, calibrated according the average genetic variance across all simulated BPFs and traits (*see Material and Methods* for details). The x-axis indicates whether h^2_{observed} in the training set of size N_{train} was (i) calculated directly from the variance of true breeding values and phenotypic values of training individuals ("True") or (ii) estimated via REML ("Estimated").

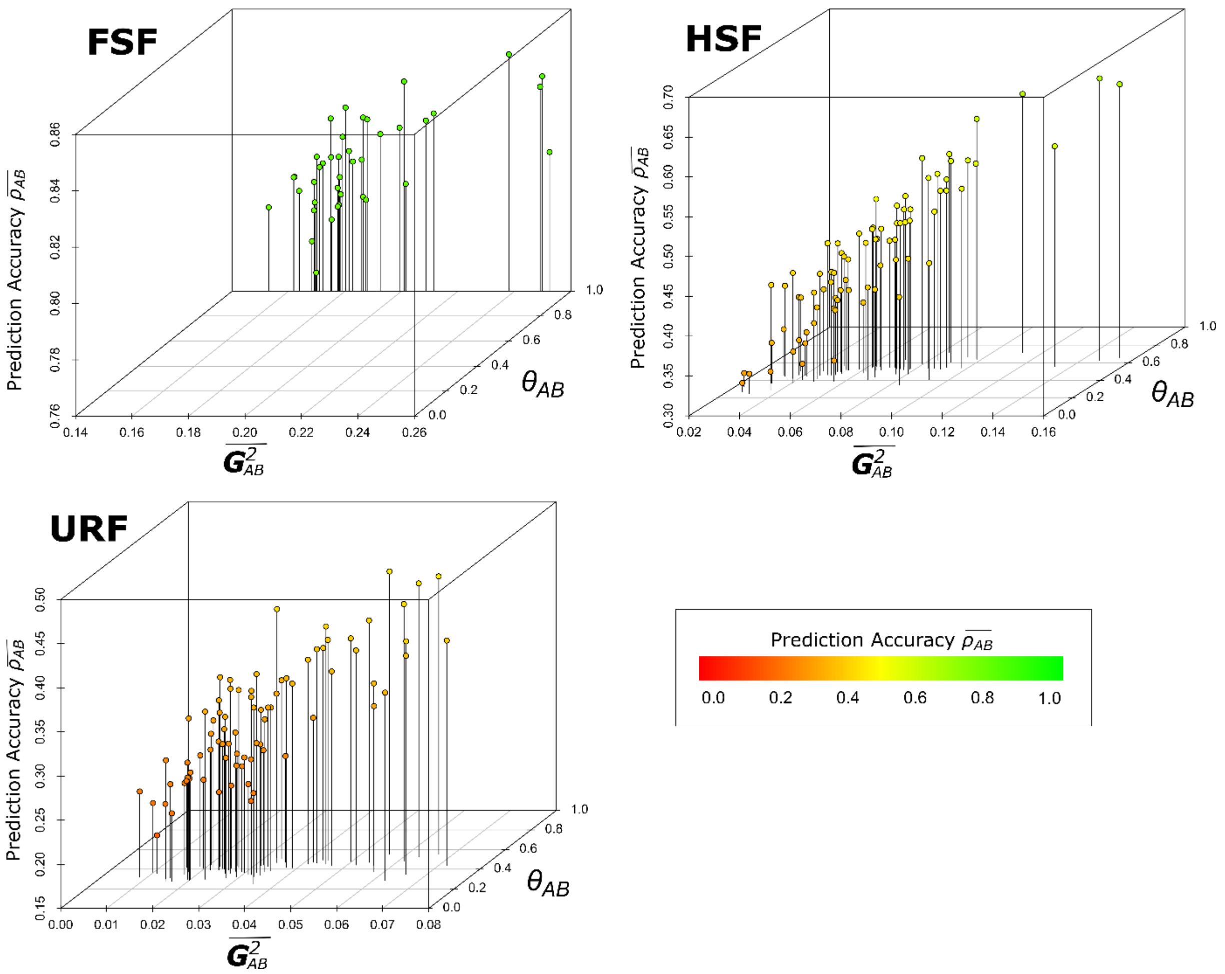

Figure S7 Means $\overline{\rho_{AB}}$ of prediction accuracy (averaged across traits T) on the z-axis, plotted against $\overline{\boldsymbol{G}_{AB}^2}$, the mean of the squared elements of the genomic matrix $\boldsymbol{G}_{AB}$ between doubled-haploid lines in the prediction set A and the training set B on the x-axis, and against the proportion of polymorphic loci in A that also segregate in the B (θ_{AB}) on the y-axis. Results are shown for a random sample of 80 full-sib families (FSF, $A = B$), 160 half-sib families (HSF) and 160 unrelated families (URF). Scales of the x-axis and z-axis were allowed to differ between plots for better visibility. Parents of BPFs were sampled from ancestral population *Elite* and genotypes at SNP markers were used to calculate the genomic relationship matrix $\boldsymbol{G}$. Results refer to $N_{train} = 100$ and $h^2 = 0.6$.

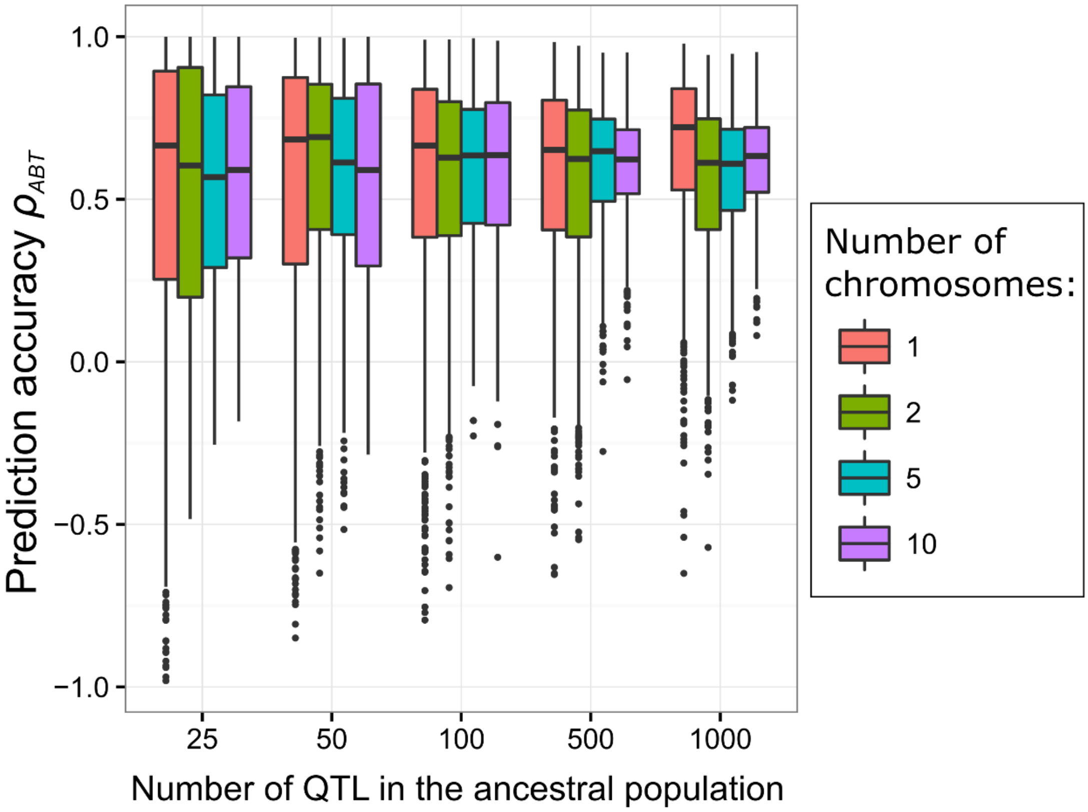

Figure S8 Boxplots of empirical prediction accuracies ρ_{ABT} of GBLUP using a QTL-derived relationship matrix, for a fixed set of doubled-haploid lines in one predicted family (BPF_{pred}) A and one training family ($\text{BPF}_{\text{train}}$) B (half-sibs) for different numbers of chromosomes and QTL. Results refer to parents sampled from ancestral population *Elite*, QTL effects sampled from a Gamma distribution $\Gamma(0.4, 1.66)$, $N_{train} = 250$, and $h^2 = 1$.

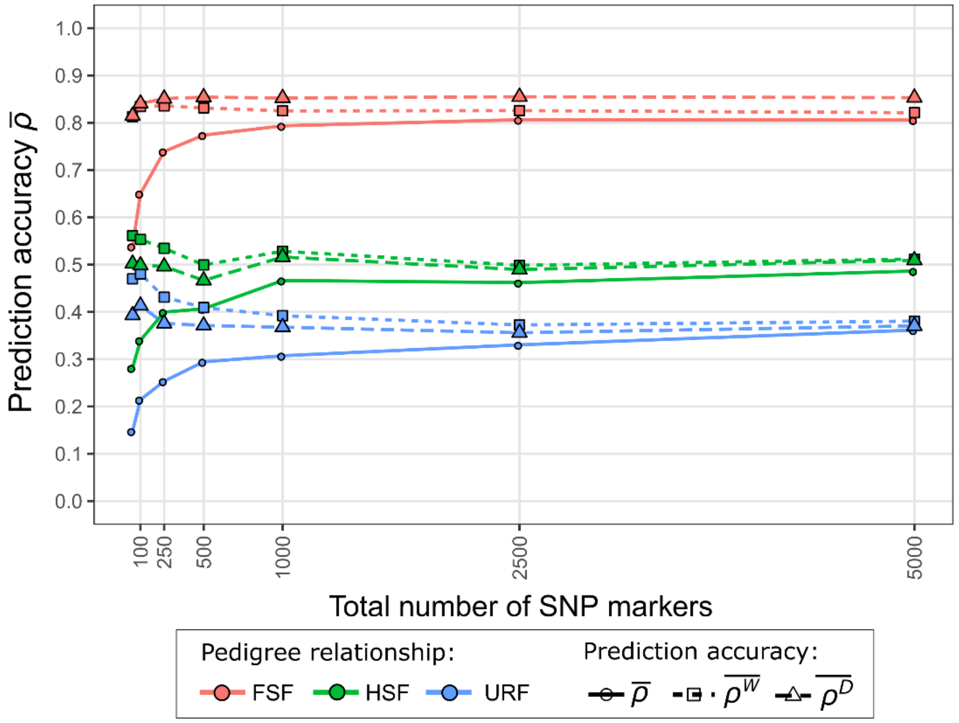

Figure S9 Comparison of mean values of empirical prediction accuracy $\bar{\rho}$ (averaged across traits and BPFs) and deterministic prediction accuracies $\overline{\rho^W}$ and $\overline{\rho^D}$ in biparental families (BPFs) of doubled-haploid lines for different numbers of SNP markers (50, 100, 250, 500, 1000, 2500, 5000). Parents of BPFs were sampled from ancestral population *Elite* and genotypes at SNP markers were used to calculate the genomic relationship matrix $\boldsymbol{G}$. Results are shown for the three pedigree relationships (full-sib*,* FSF*;* half-sib, HSF*;* unrelated family, URF) between the predicted family (BPF_{pred}) A and the training family ($\text{BPF}_{\text{train}}$) B and refer to $N_{train} = 100$ and $h^2 = 0.6$.

Tables – Supplemental Information

Table S1 List of abbreviations

Abbreviation	Meaning
GP	Genomic prediction
PA	Prediction accuracy
DH	Doubled-haploid
BPF	Biparental family
BPF_{train}	BPF from which the training set is sampled
BPF_{pred}	BPF from which the prediction set is sampled
FSF	Full-sib family
HSF	Half-sib family
URF	Unrelated family
GBLUP	Genomic best linear unbiased prediction
QTL	Quantitative trait locus
SNP	Single nucleotide polymorphism
LD	Linkage disequilibrium
GEBV	Genomic-estimated breeding value
TBV	True breeding value
CSSE	Chromosome segment substitution effect

Table S2 Degrees of freedom (d.f.) for estimation of variance components of the random factors analyzed in this study.

Pedigree relationship between predicted family A and training family B	Random factor	d.f.	value
FSF (full-sib family)	T	$t-1$	49
	A	$a-1$	49
	$A:R$	$a(r-1)$	98
	$A \times T$	$(t-1)\,(a-1)$	2,401
	$(A:R) \times T$	$(t-1)\,a(r-1)$	4,900
	**Total**	$tar-1$	7,499
HSF (half-sib family) or URF (unrelated family)	T	$t-1$	49
	A	$a-1$	49
	$A:B$	$a(b-1)$	1200
	$A:B:R$	$ab(r-1)$	2,500
	$A \times T$	$(t-1)\,(a-1)$	2,401
	$(A:B) \times T$	$(t-1)\,a(b-1)$	58,800
	$(A:B:R) \times T$	$(t-1)\,ab(r-1)$	122,500
	**Total**	$tabr-1$	187,499

Table S3 Overall mean of empirical prediction accuracy ($\bar{\rho}$) with standard deviations (SD) for different combinations of factors analyzed in our study. Results are shown for to the three pedigree relationships (full-sib, FSF; half-sib, HSF; unrelated family, URF) between the predicted family (BPF_{pred}) A and the training family (BPF_{train}) B, for different values of N_{train} and h^2. Parents of biparental families were sampled from ancestral population *Elite* or *Landrace* and genotypes at SNP markers were used to calculate the genomic relationship matrix $\boldsymbol{G}$.

			$\bar{\rho} \pm$ SD		
Landrace	h^2	N_{train}	FSF	HSF	URF
Elite	0.3	25	0.43 $\pm$ 0.20	0.24 $\pm$ 0.23	0.16 $\pm$ 0.25
		100	0.66 $\pm$ 0.14	0.37 $\pm$ 0.22	0.26 $\pm$ 0.23
		250	0.78 $\pm$ 0.09	0.45 $\pm$ 0.21	0.32 $\pm$ 0.22
	0.6	25	0.57 $\pm$ 0.17	0.32 $\pm$ 0.23	0.22 $\pm$ 0.24
		100	0.79 $\pm$ 0.09	0.45 $\pm$ 0.20	0.32 $\pm$ 0.22
		250	0.87 $\pm$ 0.05	0.51 $\pm$ 0.19	0.37 $\pm$ 0.21
	1	25	0.75 $\pm$ 0.11	0.42 $\pm$ 0.21	0.30 $\pm$ 0.23
		100	0.94 $\pm$ 0.03	0.55 $\pm$ 0.18	0.42 $\pm$ 0.20
		250	0.97 $\pm$ 0.01	0.59 $\pm$ 0.17	0.47 $\pm$ 0.20
Landrace	0.3	25	0.41 $\pm$ 0.20	0.23 $\pm$ 0.23	0.14 $\pm$ 0.23
		100	0.64 $\pm$ 0.13	0.35 $\pm$ 0.22	0.23 $\pm$ 0.23
		250	0.77 $\pm$ 0.09	0.43 $\pm$ 0.20	0.28 $\pm$ 0.22
	0.6	25	0.55 $\pm$ 0.17	0.30 $\pm$ 0.22	0.19 $\pm$ 0.23
		100	0.78 $\pm$ 0.09	0.43 $\pm$ 0.20	0.28 $\pm$ 0.22
		250	0.87 $\pm$ 0.05	0.49 $\pm$ 0.20	0.33 $\pm$ 0.21
	1	25	0.73 $\pm$ 0.11	0.40 $\pm$ 0.21	0.26 $\pm$ 0.22
		100	0.93 $\pm$ 0.03	0.53 $\pm$ 0.18	0.37 $\pm$ 0.20
		250	0.97 $\pm$ 0.01	0.57 $\pm$ 0.17	0.43 $\pm$ 0.20
